# Clarifying the Implicit Assumptions of Two-Wave Mediation Models *via* the Latent Change Score Specification: An Evaluation of Model Fit Indices

**DOI:** 10.3389/fpsyg.2021.709198

**Published:** 2021-09-06

**Authors:** Matthew J. Valente, A. R. Georgeson, Oscar Gonzalez

**Affiliations:** ^1^Center for Children and Families, Department of Psychology, Florida International University, Miami, FL, United States; ^2^Department of Psychology and Neuroscience, University of North Carolina at Chapel Hill, Chapel Hill, NC, United States

**Keywords:** longitudinal mediation, latent change scores, goodness-of-fit, mediation, equivalence testing

## Abstract

Statistical mediation analysis is used to investigate mechanisms through which a randomized intervention causally affects an outcome variable. Mediation analysis is often carried out in a pretest-posttest control group design because it is a common choice for evaluating experimental manipulations in the behavioral and social sciences. There are four different two-wave (i.e., pretest-posttest) mediation models that can be estimated using either linear regression or a Latent Change Score (LCS) specification in Structural Equation Modeling: Analysis of Covariance, difference and residualized change scores, and a cross-sectional model. Linear regression modeling and the LCS specification of the two-wave mediation models provide identical mediated effect estimates but the two modeling approaches differ in their assumptions of model fit. Linear regression modeling assumes each of the four two-wave mediation models fit the data perfectly whereas the LCS specification allows researchers to evaluate the model constraints implied by the difference score, residualized change score, and cross-sectional models *via* model fit indices. Therefore, the purpose of this paper is to provide a conceptual and statistical comparison of two-wave mediation models. Models were compared on the assumptions they make about time-lags and cross-lagged effects as well as statistically using both standard measures of model fit (χ^2^, RMSEA, and CFI) and newly proposed *T*-size measures of model fit for the two-wave mediation models. Overall, the LCS specification makes clear the assumptions that are often implicitly made when fitting two-wave mediation models with regression. In a Monte Carlo simulation, the standard model fit indices and newly proposed *T*-size measures of model fit generally correctly identified the best fitting two-wave mediation model.

## Introduction

The questions asked in analyses of randomized interventions are inherently about change. For example, Kunze et al. (2019) assessed if imagery rescripting, a treatment for nightmare disorder, caused a change in nightmare distress *via* changing the participant's self-efficacy. Generally, interventionists might ask if the program was able to change a health outcome (e.g., nightmare distress), if the program components successfully changed the mechanism (e.g., self-efficacy), or how a change in a mechanism led to a change in the health outcome. Statistical mediation analysis is used to investigate mechanisms through which a randomized intervention causally affects an outcome variable (Lazarsfeld, 1955; Baron and Kenny, 1986; MacKinnon, 2008) and is often carried out in a pretest-posttest control group designs to address questions of change. While there are many ways in which to investigate mediating mechanisms over time [see for example, MacKinnon (2008, Chapter 8), Vuorre and Bolger (2018), and Montoya (2019)], we focus on the pretest-posttest control group design because it is a common design for evaluating experimental manipulations in the behavioral and social sciences.

Traditionally, researchers use several ways to represent the change across time of mediators and outcomes in the statistical mediation model (MacKinnon, 2008, Chapter 8). For example, researchers could use a difference score, which is the difference between the score at pretest and the score at posttest. A second approach is to use a residualized change score, which is the residual left over after the posttest score is regressed on the pretest score. At face value, difference scores and residualized change scores directly address the question of change—they represent how a variable changed from pretest to posttest—thus, they remain popular approaches in the social sciences despite having some drawbacks [see the discussion section for more details; see MacKinnon et al. (1991), Jansen et al. (2013), Cederberg et al. (2016), Silverstein et al. (2018), Kunze et al. (2019), for difference score mediation models, and Miller et al. (2002), Slee et al. (2008), Quilty et al. (2008), Reid and Aiken (2013), for residualized change score mediation models]. A third approach is the ANCOVA model which treats pretest measures of the mediator and outcome as covariates when analyzing the posttest mediator-outcome relation (MacKinnon, 1994; MacKinnon et al., 2001; Schmiege et al., 2009; Jang et al., 2012). Finally, it is also possible to estimate a cross-sectional model which ignores the pretest measures. The cross-sectional model is discussed because it is one of the four possible models that can be fit with two-waves of data. The difference score, residualized change score, and cross-sectional models make very stringent assumptions about the relationship between the pretest and posttest measures for both the mediator and the outcome (Valente and MacKinnon, 2017). These assumptions are rarely evaluated, and we suspect that this lack of evaluation is because researchers did not have the tools or guidance to do so.

Statistical mediation models with difference scores, residualized change scores, or a cross-sectional model can be parameterized using a Latent Change Score (LCS; McArdle, 2001, 2009) specification (Valente and MacKinnon, 2017). Using the LCS specification, it can be shown that statistical mediation models with difference scores or residualized change scores are *nested* within the ANCOVA model. As such, the LCS specification provides researchers with a venue to assess model fit which is not possible using traditional approaches for estimating these models (e.g., regression modeling). This is an important finding because it allows researchers to move beyond questions asked within a Null-Hypothesis Significance Testing (NHST) framework (e.g., which two-wave mediation models produced statistically significant mediated effect estimates?) and benefit from model-based thinking (e.g., which model best describes the psychological process under investigation and which model best fits the data?; Rodgers, 2010). However, despite the promise of using this framework to assess the fit of these various models, the performance of traditional (West et al., 2012) and newly-developed fit statistics (Yuan et al., 2016) to assess the fit for these models of change must first be evaluated. While statistical properties (i.e., Type 1 error rates, statistical power, confidence interval coverage, and relative bias) of these models of change were investigated in previous research (Valente and MacKinnon, 2017), the models of change were not compared under conditions that explicitly match the constraints implied by each model and the performance of model fit indices was not investigated.

Therefore, the goals of this paper are to demonstrate the advantage of the LCS framework over the regression framework for two-wave mediation models, assess the performance of model fit indices for these models, and provide guidance for applied researchers in how to evaluate the performance of model fit indices for assessing the adequacy of the model constraints that are implied by each of these models. This paper starts with an overview of the two-wave mediation model, followed by an overview of the approach of fitting each model using an LCS specification. Next, the χ^2^ test is described in general and for each specific nested model followed by additional model fit statistics. Then, results are presented from a simulation study on the performance of model fit indices to evaluate the fit of different models of change. Finally, an empirical example is presented to demonstrate the advantages of fitting the models using the LCS specification compared to regression modeling.

### Two-Wave Mediation Models

The simplest longitudinal mediation model that can be used to estimate the mediated effect of a randomized intervention on an outcome is the two-wave mediation model. The two-wave mediation model consists of pretest (or baseline) measures of the mediator and outcome variable collected prior to units being randomized to levels of an intervention and posttest measures of the mediator and outcome variables after units have been randomized to levels of an intervention ^1^.

Three popular two-wave mediation models to examine change include ANCOVA, difference scores, and residualized change scores. We recommend readers to review MacKinnon (2008, Chapter 8) for differences in these models. Another possible model that researchers could investigate with two-waves of data is the cross-sectional mediation model, in which researchers ignore the measures at pretest altogether. In other words, the cross-sectional mediation model is not a model of change, but we describe it to understand the consequences of ignoring pretest measures. Below, we describe the typical specification of these four models.

#### ANCOVA

The following three equations can be used to describe the relations among the intervention (*X*), mediator (*M*), and outcome (*Y*) variables in the two-wave mediation model (MacKinnon, 2008; Valente and MacKinnon, 2017).

(1) $$\begin{array}{r} Y_{2}=i_{1}+c_{y2x}X+e_{1} \end{array}$$

(2) $$\begin{array}{r} M_{2}=i_{2}+\;a_{m2x}X+s_{m2m1}M_{1}+b_{m2y1}Y_{1}+e_{2} \end{array}$$

(3) $$\begin{array}{r} Y_{2}=i_{3}+{c^{\prime }}_{y2x}X+s_{y2y1}Y_{1}+b_{y2m1}M_{1}+b_{y2m2}M_{2}+e_{3} \end{array}$$

The ANCOVA estimate of the mediated effect in this model can be equivalently estimated as the product *a*_*m*2*x*_*b*_*y*2*m*2_ from Equations (2, 3) or the difference *c*_*y*2*x*_- *c'*_2*yx*_ from Equations (1, 3).

#### Difference Score Model

Equations (4, 5) represent regression equations using difference scores for the mediator variable and outcome variable, respectively. The difference score for the mediator is Δ_*M*_ = *M*_2_ − *M*_1_. The difference score for the outcome is Δ_*Y*_ = *Y*_2_ − *Y*_1_. These difference scores represent change on the mediator and outcome from pretest, respectively.

(4) $$\begin{array}{r} \Delta_{M}=i_{6}+a_{\Delta }X+e_{6} \end{array}$$

(5) $$\begin{array}{r} \Delta_{Y}=i_{7}+{c^{\prime }}_{\Delta }X+b_{\Delta }\Delta_{M}+e_{7} \end{array}$$

The mediated effect is estimated by computing the product of *a*_Δ_ coefficient from Equation (4) and *b*_Δ_ coefficient from Equation (5) (*a*_Δ_*b*_Δ_) which is the effect of *X* on change in *Y* through its effect on change in *M*.

#### Residualized Change Score Model

Equations (6, 7) represent regression equations using residualized change scores for the mediator variable and the outcome variable, respectively. The residualized change score for the mediator variable is *R*_*M*_ = *M*_2_ − *E*[*M*_2_|*M*_1_] which is the change in predicted scores on the mediator variable measured at posttest subtracted from observed scores on the mediator variable measured at posttest. The residualized change score for the outcome variable is *R*_*Y*_ = *Y*_2_ − *E*[*Y*_2_|*Y*_1_] which is the change in predicted scores on the outcome variable measured at posttest subtracted from observed scores on the outcome variable at posttest.

(6) $$\begin{array}{r} R_{M}=i_{8}+a_{R}X+e_{8} \end{array}$$

(7) $$\begin{array}{r} R_{Y}=i_{9}+{c^{\prime }}_{R}X+b_{R}R_{M}+e_{9} \end{array}$$

The mediated effect is estimated by computing the product of *a*_*R*_ coefficient from Equation (6) and *b*_*R*_ coefficient from Equation (7) (*a*_*R*_*b*_*R*_) which is the effect of *X* on the residual change in *Y* through its effect on the residual change in *M*.

#### Cross-Sectional Model

The cross-sectional model is the simplest model because it does not take into account the pretest measures of the mediator and outcome and therefore does not address a question of change across time. Equation (8) represents the relation between the treatment variable and the posttest mediator (*a*_*m*2*x*_) and Equation (9) represents the relation between the treatment variable and the posttest outcome (*c'*_*y*2*x*_) adjusted for the posttest mediator and the relation between the posttest mediator and the posttest outcome (*b*_*y*2*m*2_) adjusted for the treatment.

(8) $$\begin{array}{r} M_{2}=i_{4}+\;a_{m2x}X+e_{4} \end{array}$$

(9) $$\begin{array}{r} Y_{2}=i_{5}+{c^{\prime }}_{y2x}X+b_{y2m2}M_{2}+e_{5} \end{array}$$

The cross-sectional mediated effect is estimated by computing the product of *a*_*m*2*x*_ coefficient from Equation (8) and *b*_*y*2*m*2_ coefficient from Equation (9) (*a*_*m*2*x*_*b*_*y*2*m*2_) which is the effect of *X* on *Y*_2_ through its effect on *M*_2_ not adjusted for pretest measures, *M*_1_ and *Y*_1_.

### Latent Change Score Specification for Two-Wave Mediation Models

**Figure 1:**
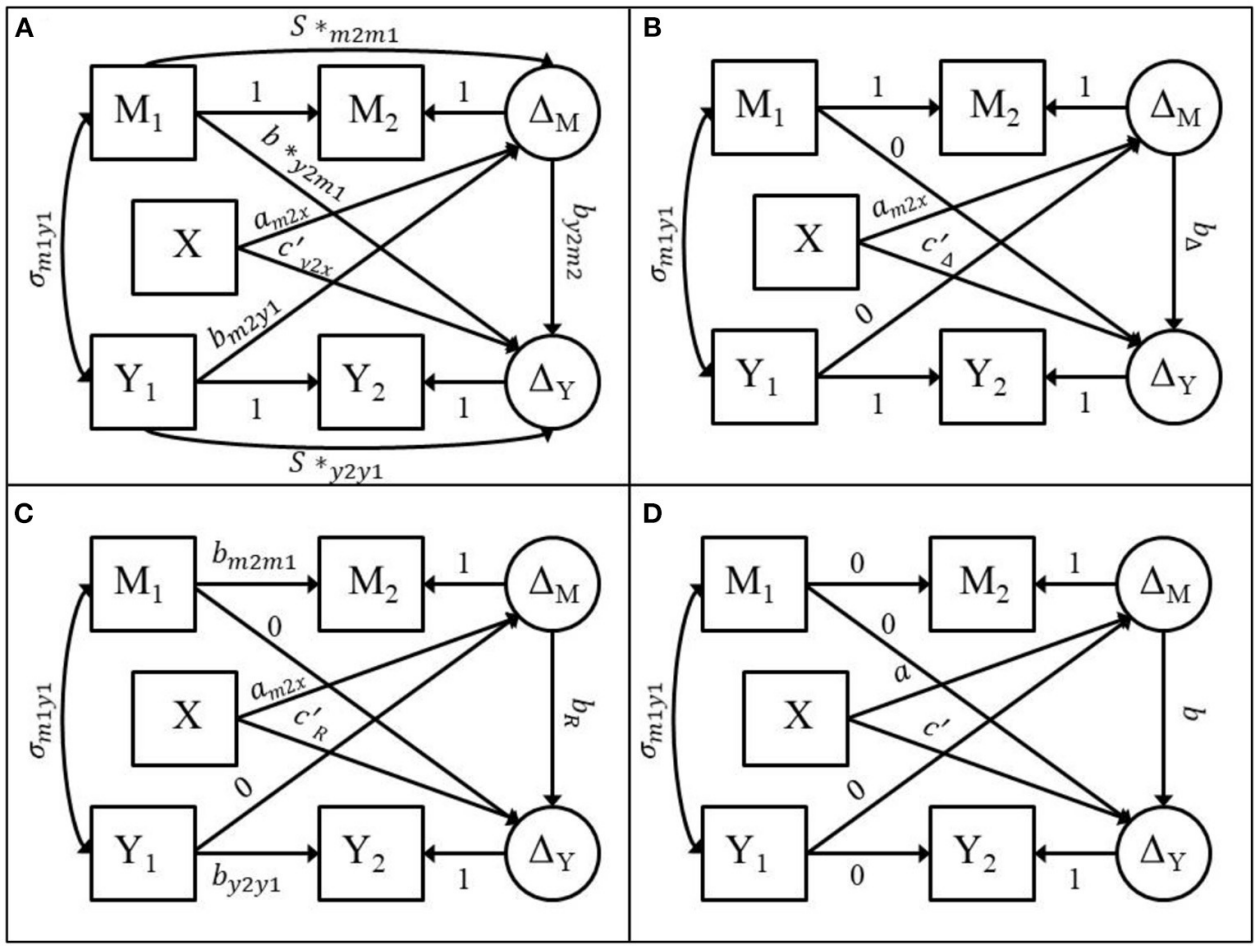
Adapted from Valente and MacKinnon (2017). **(A)** LCS specification of the ANCOVA two-wave mediation model. **(B)** LCS specification of the difference score model. **(C)** LCS specification of the residualized change score model. **(D)** LCS specification of the cross-sectional model.

LCS specification is a SEM approach to modeling longitudinal data that can represent simple and dynamic change over time with either manifest or latent measures of a time-dependent outcome (McArdle, 2001, 2009; Grimm et al., 2017). For the two-wave mediation model, all four two-wave models previously mentioned can be fitted with the LCS specification (see Figure 1). The two-wave mediation model displayed in Figure 1 contains 20 free parameters across the mean and covariance structure. Figure 1A displays the full ANCOVA model, 1B displays the difference score model, 1C displays the residualized change score model, and 1D displays the cross-sectional model. The LCS specification for each of the two-wave mediation models can be used to evaluate the assumptions encoded by the difference score, residualized change score, and cross-sectional models *via* model fit indices.

The full ANCOVA model is estimated by creating a latent change score for the mediator (Δ*M*) which has a loading on *M*_2_ that is fixed to 1.0 while the mean and variance of Δ*M* are freely estimated. Next, the path from *M*_1_ to *M*_2_ is fixed to 1.0, the mean and variance of *M*_2_ are constrained to zero, and the mean and variance of *M*_1_ are freely estimated. The same steps are followed to compute the latent change score for the outcome variable (Δ*Y*). The covariances between *M*_1_, *Y*_1_, and *X* are freely estimated. Δ*M* is then regressed on *X, M*_1_, and *Y*_1_and Δ*Y* is regressed on *X*, Δ*M, M1*, and *Y*_1_. The full ANCOVA model is a saturated model with zero degrees of freedom (*df*).

The difference score model is obtained by constraining the s*_m2*m*1_, s*_y2*y*1_, b*_y2*m*1_, and *b*_*m*2*y*1_ parameters in Figure 1A to zero as shown in Figure 1B. The difference score model has four *df*. The residualized change score model is obtained by constraining the b*_y2*m*1_, and *b*_*m*2*y*1_ parameters in Figure 1A to zero and the parameters s*_m2*m*1_ and s*_y2*y*1_, in Figure 1A to *b*_*m*2*m*1_ and *b*_*y*2*y*1_, respectively as shown in Figure 1C. *b*_*m*2*m*1_ is the regression coefficient estimate from a linear regression of *M*_2_ on *M*_1_ and *b*_*y*2*y*1_ is the regression coefficient estimate from a linear regression of *Y*_2_ on *Y*_1_. The residualized change score model has four *df*. The cross-sectional model is obtained by constraining the $s_{m2m1}^{*}$, $s_{y2y1}^{*}$, $b_{y2m1}^{*}$, *b*_*m*2*y*1_ parameters and the paths from *M*_1_ to *M*_2_ and from *Y*_1_ to *Y*_2_ in Figure 1A to zero as shown in Figure 1D. The cross-sectional model has four *df*. These models can be fitted using any SEM software. Because these models are fitted using SEM, we can use the model fit indices to evaluate the adequacy of the model in this dataset.

It was demonstrated how these four models can be estimated using a Latent Change Score (LCS) specification and that the difference score, residualized change score, and cross-sectional models make strict assumptions about the stability of the mediator and outcome variables and the cross-lagged paths from the pretest measures of the mediator and the outcome to the posttest measures of the mediator and the outcome. These models are therefore nested within the ANCOVA model (Valente and MacKinnon, 2017). The implication is that the difference score, residualized change score, and cross-sectional models are *not* fully saturated models that fit the data perfectly.

In summary, the LCS specification provides researchers with two advantages over regression modeling. First, the LCS specification helps researchers clarify the assumptions they are making regarding each model of change because these assumptions are encoded in the LCS path diagrams thus resulting in a clearer understanding of the theoretical implications of each model. Second, the LCS specification provides the added benefit of supplementing theoretical considerations of model choice with fit statistics. Below, we describe how model fit is evaluated.

### Evaluating the Fit of Different Models of Change

There are several fit statistics that researchers could use to evaluate fit. Some of these fit statistics include: the χ^2^ goodness of fit statistic, the Comparative Fit Index (CFI; Bentler, 1990), the Root Mean Square Error of Approximation (RMSEA; Steiger, 1989), and the newly proposed *T*-size CFI and RMSEA (Yuan et al., 2016). Performance of the standard fit indices has been investigated in LCS models in the context of measurement non-invariance (Kim et al., 2020) and when testing the performance of the fit indices in selecting the nested autoregressive cross-lagged factor model (Usami et al., 2015, 2016). The performance of these standard fit indices and the *T*-size fit indices for selecting alternative two-wave mediation models has never been investigated.

#### The **χ**^**2**^ Test

Model χ^2^ goodness of fit tests or simply, χ^2^ tests, can be used to test the fit of a statistical model or used to compare the fit of two competing models such that one model is nested within another model (Bentler and Bonett, 1980; Bollen, 1989; West et al., 2012). A model is considered nested within a full model if it is possible to estimate the parameters of the nested model by constraining parameters of a full model to zero, effectively removing them from the model. As demonstrated above, the difference score model in Figure 1B is nested within the ANCOVA model in Figure 1A because constraining the $s_{m2m1}^{*}$, $s_{y2y1}^{*}$, $b_{y2m1}^{*}$, and *b*_*m*2*y*1_ parameters to zero results in the difference score model with four *df*. Because the ANCOVA model is fully saturated and fits the data perfectly, the χ^2^ tests of the nested models are simply the χ^2^ goodness of fit tests. Therefore, the χ^2^ test can then be used to test the null hypothesis that the difference score model fits the data perfectly assuming a χ^2^distribution with *df* equal to four. Therefore, rejecting the null hypothesis provides evidence that the difference score model does not fit the data perfectly. For example, we might fit the difference score model that is displayed in Figure 1B and observe χ^2^ = 7.5, *df* = 4. The critical value for a chi-square distribution with 4 degrees of freedom at *p*-value = 0.05 is 9.488. Therefore, we fail to reject the null hypothesis that the difference score model fits the data perfectly, thus providing justification to fit the difference score model.

Failing to reject the null hypothesis provides statistical evidence that estimating the extra four parameters in the ANCOVA model does not result in a significantly better fitting model as compared to the fit of the simpler, more parsimonious difference score model (i.e., simpler in terms of less estimated parameters). In other words, the psychological phenomenon characterized by this two-wave mediation model can be explained equally well using a simpler model with fewer estimated parameters compared to a more complicated model with more estimated parameters. The χ^2^ test is a test of perfect model fit which may be unrealistic in practice (MacCallum et al., 2001). Therefore, it is important to investigate how each model fits the data by using other indexes of model fit.

#### CFI and RMSEA

The Comparative Fit Index (CFI) is a goodness of fit index that measures how well model-implied covariances match the observed covariances in the data (Bentler, 1990). Higher values of the CFI indicate better fit than lower values of the CFI (Bentler, 1990; West et al., 2012). The Root Mean Square Error of Approximation (RMSEA) is a badness of fit index that measures how poorly model-implied covariances match the observed covariances in the data (Steiger and Lind, 1980; Steiger, 1989, 2016). Lower values of the RMSEA indicate better fit than higher values of the RMSEA (Steiger and Lind, 1980; Steiger, 1989, 2016; West et al., 2012). Both the CFI and the RMSEA have cut-off values that are used as a rule-of-thumb to determine at which values of the respective fit indexes a model is considered to fit the data well.

#### Equivalence Testing and T-Size Measures

While the null hypothesis of the χ^2^test can provide information that a model does not fit perfectly and the CFI and RMSEA can provide information about the goodness of model fit and the badness of model fit, respectively, neither of these indexes of model fit provide information about *endorsing* the null hypothesis for model fit. Ideally, there would be a measure that could provide some level of confidence that the model fit is within a specified range of the null hypothesis. In other words, the rejection of the standard null hypothesis of model fit will tell us the model does not fit perfectly but failure to reject the null does not tell us that the model *does* fit perfectly. Recent papers by Yuan et al. (2016) and Marcoulides and Yuan (2017, 2020) provide such measures for SEMs *via equivalence testing*.

The goal of equivalence testing is to *endorse* a model under the standard null hypothesis instead of *rejecting* a model under the standard null hypothesis. In order to conduct equivalence testing, a minimum tolerable size of model misspecification (ε_*t*_; i.e., the *T*-size) corresponding to the observed χ^2^-test statistic must be determined. The main goal of equivalence testing is to accurately reject a model. This happens when the observed χ^2^-test statistic falls within a specific interval between zero and a left-tail critical value with cumulative probability equal to α from a non-central χ^2^ distribution with a specified level of misspecification and *df* equal to the observed model *df*. The rejection of the null hypothesis at α = 0.05, implies the model misspecification is within a tolerable size. This is opposed to standard null hypothesis significance testing which tests if the observed χ^2^-test statistic falls above a right-tail critical value with cumulative probability equal to 1—α from a central χ^2^ distribution with *df* equal to the observed model *df* [for a complete treatment and details on how the significance regions are calculated, see Yuan et al. (2016)]. In keeping with the literature on equivalence testing in SEM, the tolerable size of misspecification can be transformed into a *T*-size RMSEA, or CFI value. Regarding the RMSEA, the *T*-size measures are interpreted at α = 0.05 as “we are 95% confident that the misspecification is *X-*units as measured by the RMSEA” and the *T*-size CFI is interpreted as “we are 95% confident that the population CFI is above *X*” (Marcoulides and Yuan, 2017).

Because equivalence testing results in new *T*-size RMSEA and CFI values that take into account a specified level of model misspecification, it is not appropriate to compare these *T*-size RMSEA and CFI values to the standard cut-off values. To remedy this, Yuan et al. (2016) derived adjusted cut-off values for the *T*-size RMSEA and CFI values for which the *T*-size RMSEA and CFI values can be compared, respectively. The adjusted RMSEA cut-off values are estimated based on the observed sample size and model degrees of freedom and are therefore estimated for each sample and each model being fit to the data. The interpretation of the adjusted cut-off values is therefore conditional on our specified level of model misspecification. In other words, our model may have excellent, close, fair, mediocre, or poor fit given the specified level of misspecification.

Regarding the CFI, equivalence testing compares the fit of the observed model to the misfit of the baseline model for the CFI. The adjusted cut-off values for the CFI are a function of the sample size, model degrees of freedom, number of predictors, and baseline model degrees of freedom. The adjusted CFI cut-off values are therefore estimated for each sample and each model being fit to the data. Similar to the adjusted cut-off values for the RMSEA, the interpretation of the adjusted cut-off values for the CFI are conditional on our specified level of model misspecification. In other words, our model may have excellent, close, fair, mediocre, or poor fit given the specified level of misspecification.

### Present Study

The LCS specification allows researchers to test the adequacy of the model constraints imposed by the difference score, residualized change score, and cross-sectional models. This is an advantage over the regression-based approach for these models because the regression-based approach does not have any way of evaluating how the model constraints may impact the fit of the models to the observed data. Since the model fit can be assessed for each of these models, researchers can use fit indexes in SEM to evaluate the appropriateness of these model constraints for their observed data. Therefore, it is important to know how the χ^2^ test, CFI, and RMSEA, along with the equivalence measures, will perform when evaluating the fit of these two-wave mediation models (i.e., do the indices support or reject model fit when they should).

The purpose of the simulation study is to demonstrate which factors of the two-wave mediation model are important predictors of the Type 1 error and power of the χ^2^ test when used to test the difference score model, the residualized change score model, and the cross-sectional model. There are three main hypotheses for the simulation study that are driven from the constraints that are made to fit each of these models: (1). When stability = 1.00 and cross-lags = 0 (i.e., the true model is the difference score model) the null hypothesis of the χ^2^ test assessing the fit of the difference score model should not be rejected; (2). When both cross-lagged paths = 0 (i.e., the residualized change score model is the true model), the null hypothesis of the χ^2^ test assessing the fit of the residualized change score model should not be rejected; (3).When stability = 0 and cross-lags = 0 (i.e., the cross-sectional model is the true model), the null hypothesis of the χ^2^ test assessing the fit of the cross-sectional model should not be rejected. Further, it is expected that the CFI, *T*-size CFI, RMSEA, and *T*-size RMSEA values will indicate close or excellent fit when the respective model assumptions are met. Further, we have an empirical illustration to highlight the differences between the estimation of the models of change using regression and the LCS specification.

## Simulation Study

### Method

SAS 9.4 was used to conduct Monte Carlo simulations. The following equations represent the linear regression model used to generate the data where *x* is an observed value of *X* and $\tilde{x}$ is the sample median.

(10) $$\begin{array}{r} X\;\sim N\left(0,1\right):\left(x\geq \tilde{x}\right)=1;\left(x<\tilde{x}\right)=0 \end{array}$$

(11) $$\begin{array}{r} M_{1}\sim N\left(0,1\right) \end{array}$$

(12) $$\begin{array}{r} Y_{1}=b_{y1m1}M_{1}+e_{1} \end{array}$$

(13) $$\begin{array}{r} M_{2}=a_{m2x}X+b_{m2y1}Y_{1}+s_{m2m1}M_{1}+e_{2} \end{array}$$

(14) $$\begin{array}{r} Y_{2}={c^{\prime }}_{y2x}X+b_{y2m1}M_{1}+b_{y2m2}M_{2}+s_{y2y1}Y_{1}+e_{3} \end{array}$$

The factors varied were: sample size (*N* = 50, 100, 200, 500); effect size of the *a*_*m*2*x*_ (0, 0.14, 0.39, 0.59), *b*_*y*2*m*2_ (0, 0.14, 0.39, 0.59), and *c'*_*y*2*x*_ (0, 0.39) paths; effect size of the *Y*_2_ cross-lagged path *b*_*y*2*m*1_ (0, 0.50) and *M*_2_ cross-lagged path *b*_*m*2*y*1_ (0, 0.50); stability of the mediating variable (*s*_*m*2*m*1_) and outcome variable (*s*_*y*2*y*1_) (0, 0.30, 1.00); and relation between *M*_1_ and *Y*_1_ (0, 0.50). These factors were varied to test hypotheses 1 – 3. All residual terms (*e*_1_, *e*_2_, and *e*_3_) had a standard deviation of one, were uncorrelated with each other and the predictors. The effect sizes were chosen to reflect approximately small, medium, and large effect sizes (Cohen, 1988). A full factorial design produced 3,072 conditions, each with 1,000 replications. All models were fit using SAS PROC CALIS. The *T*-size measures of the CFI and RMSEA were obtained using the R function provided by Yuan et al. (2016).

The raw data were analyzed using analysis of variance (ANOVA). The dataset contained 3,072,000 observations consisting of 1,000 replications for each of the 3,072 conditions. All significant main effects and interactions with semi-partial eta-squared values of 0.005 or greater (rounded to the third decimal place) were considered important and reported in the Supplementary Materials along with simulation results for additional fit indexes (SRMR, AIC, and BIC). The ANOVA was used to determine the pattern of results described in the proceeding results section. The CFI and RMSEA values of the ANCOVA model were not reported because the ANCOVA model is a saturated model with zero degrees of freedom therefore it fits the data perfectly. Type 1 error rates of the χ^2^ tests were deemed acceptable if they fell within the robustness interval [0.025, 0.075] (Bradley, 1978). Sample size was not a significant predictor of the performance of the χ^2^ tests, CFI, or RMSEA therefore all results for these fit indices were collapsed across sample sizes.

### χ^2^ Test Results

#### Difference Score Model

The Type 1 error rate of this χ^2^ test can be assessed when stability = 1.00 and both cross-lagged paths = 0. For this condition, the Type 1 error rates were within the robustness interval but only when the *b*_*y*2*m*2_ path = 0 (i.e., α = 0.057). When the *b*_*y*2*m*2_ path = 0.14 the Type 1 error rate increased to α = 0.347 and approached 1.00 as the *b*_*y*2*m*2_ path increased to 0.59. Power can be assessed when stability <1.00 and either or both cross-lagged paths > 0. When stability was <1.00 and as the *b*_*y*2*m*2_ path, *M*_2_ cross-lag, and *Y*_2_ cross-lag paths increased in magnitude, the power of the χ^2^ test to reject the null hypothesis of perfect fit for the difference score model approached 1.00.

#### Residualized Change Score Model

The Type 1 error rate of this χ^2^ test can be assessed when both cross-lagged paths = 0. For this condition, the Type 1 error rates increased as stability approached 1.00 and the *b*_*y*2*m*2_ path increased in magnitude. The condition that resulted in the lowest Type 1 error rate was when stability = 0.00, the *b*_*y*2*m*2_ path = 0.00 and both cross-lags = 0.00 (i.e., α = 0.013) but the Type 1 error rate for this condition was below the lower bound of the robustness interval. The Type 1 error rates were within the robustness interval when both cross-lags = 0.00 and stability = 0.00 and when *b*_*y*2*m*2_ = 0.59. Power can be assessed when either or both cross-lagged paths > 0. As the *M*_2_ cross-lag and *Y*_2_ cross-lag paths increased from 0.00 to 0.50, the power of the χ^2^ test to reject the null hypothesis of perfect fit for the residualized change score model approached 1.00.

#### Cross-Sectional Model

The Type 1 error rate of this χ^2^ test can be assessed when stability = 0 and both cross-lagged paths = 0. For this condition, the Type 1 error rate was within the robustness interval (i.e., α = 0.057). Power can be assessed when stability > 0 and either or both cross-lagged paths > 0. As stability, the *M*_2_ cross-lag, and the *Y*_2_ cross-lag paths increased from 0.00 to 0.50, the power of the χ^2^ test to reject the null hypothesis of perfect fit for the cross-sectional model approached 1.00.

### CFI and *T*-size CFI Results

Because the CFI and *T*-size CFI (CFI_t) results were below the conventional cut-off values when either or both the cross-lagged paths were greater than zero, the results were reported for the condition *M*_2_ cross-lag = 0 and *Y*_2_ cross-lag = 0. Both the CFI and CFI_t values were compared to their respective cut-off values. For the CFI values, the conventional cut-offs are: 0.99 – excellent; 0.95 – close; 0.92 – fair; 0.90 mediocre; <0.90 – poor. The adjusted cut-off values for the CFI_t are a function of the sample size, model degrees of freedom, number of predictors, and baseline model degrees of freedom. Because the models tested contain the same degrees of freedom, same number of predictors, and same baseline model degrees of freedom, the adjusted CFI cut-off values varied only as a function of sample size. Therefore, the CFI_t results were reported for each sample size. The adjusted CFI cut-off values for *N* = 50 were: 0.77 – excellent; 0.66 – close; 0.59 – fair; 0.54 mediocre; <0.54 – poor. The adjusted CFI cut-off values for *N* = 100 were: 0.88 – excellent; 0.79 – close; 0.73 – fair; 0.69 mediocre; <0.69 – poor. The adjusted CFI cut-off values for *N* = 200 were: 0.94 – excellent; 0.86 – close; 0.80 – fair; 0.77 mediocre; <0.77 – poor. The adjusted CFI cut-off values for *N* = 500 were: 0.97 – excellent; 0.90 – close; 0.86 – fair; 0.83 mediocre; <0.83 – poor.

#### Difference Score Model

Table 1 displays the CFI results tabled as a function of the *b*_*y*2*m*2_ path, baseline correlation, and stability. As stability increased to 1.00, the CFI increased in magnitude and approached the conventional level of excellent fit when the *b*_*y*2*m*2_ path = 0 and approached the conventional level of close fit when the *b*_*y*2*m*2_ path = 0.14. As the *b*_*y*2*m*2_ path increased in magnitude the CFI values decreased to below the conventional level of poor fit (for example, CFI = 0.781 and 0.832 for baseline correlation = 0 and baseline correlation = 0.5, respectively; see Table 1).

**Table 1:** CFI values for the difference score model (Diff), residualized change score model (Res), and cross-sectional model (Cross) for *M*_2_cross-lag = 0 and *Y*_2_ cross-lag = 0.

		**Base. Corr**.					
		**0**			**0.5**		
		**Diff**	**Res**	**Cross**	**Diff**	**Res**	**Cross**
Stability	b_*y*2*m*2_ path						
0	0	0.000	0.970	0.887	0.000	0.993	0.976
	0.14	0.000	0.974	0.918	0.001	0.993	0.977
	0.39	0.001	0.985	0.967	0.006	0.993	0.983
	0.59	0.003	0.986	0.983	0.029	0.991	0.988
0.3	0	0.014	0.990	0.474	0.048	0.995	0.711
	0.14	0.020	0.988	0.514	0.079	0.994	0.730
	0.39	0.040	0.969	0.678	0.175	0.985	0.786
	0.59	0.072	0.940	0.797	0.287	0.968	0.841
1	0	0.992	0.998	0.083	0.994	0.998	0.243
	0.14	0.982	0.989	0.098	0.986	0.993	0.277
	0.39	0.902	0.910	0.182	0.926	0.948	0.351
	0.59	0.781	0.788	0.298	0.832	0.872	0.428

*CFI values that were above the conventional cut-off for close fit are bolded and underlined. The conventional cut-off values are: 0.99 – excellent; 0.95 – close; 0.92 – fair; 0.90 mediocre; <0.90 - poor*.

Table 2 displays the *T*-size or CFI_t results tabled as a function of the *b*_*y*2*m*2_ path, baseline correlation, stability, and sample size for all models. Across all sample sizes, as stability increased to 1.00, the CFI_t values were above the adjusted cut-off value for close fit when the *b*_*y*2*m*2_ path was less than or equal to 0.14 across both values of the baseline correlation. The CFI_t values were higher when the baseline correlation = 0.50 (see Table 2).

**Table 2:** *T*-size CFI values (CFI_t) for the difference score model (Diff), residualized change score model (Res), and cross-sectional model (Cross) for *M*_2_cross-lag = 0 and *Y*_2_ cross-lag = 0.

			**Sample size**											
			**50**			**100**			**200**			**500**		
			**Diff**	**Res**	**Cross**	**Diff**	**Res**	**Cross**	**Diff**	**Res**	**Cross**	**Diff**	**Res**	**Cross**
Stability	Base. Corr.	b_*y*2*m*2_ path												
0	0	0	0.003	0.198	0.101	0.002	0.393	0.244	0.001	0.578	0.448	0.001	0.763	0.678
		0.14	0.002	0.228	0.116	0.002	0.408	0.277	0.000	0.623	0.489	0.000	0.787	0.693
		0.39	0.000	0.285	0.188	0.000	0.551	0.438	0.000	0.765	0.695	0.000	0.928	0.902
		0.59	0.000	0.408	0.357	0.000	0.706	0.678	0.000	0.882	0.872	0.000	0.961	0.958
	0.5	0	0.000	0.393	0.220	0.000	0.689	0.535	0.000	0.870	0.800	0.000	0.960	0.937
		0.14	0.000	0.418	0.249	0.000	0.701	0.563	0.000	0.873	0.813	0.000	0.963	0.942
		0.39	0.000	0.505	0.357	0.000	0.778	0.689	0.000	0.913	0.876	0.000	0.970	0.959
		0.59	0.000	0.570	0.523	0.000	0.833	0.808	0.000	0.932	0.922	0.000	0.977	0.973
0.3	0	0	0.000	0.293	0.011	0.000	0.550	0.026	0.000	0.772	0.082	0.000	0.934	0.173
		0.14	0.000	0.320	0.012	0.000	0.555	0.040	0.000	0.769	0.109	0.000	0.918	0.209
		0.39	0.000	0.327	0.041	0.000	0.552	0.110	0.000	0.758	0.227	0.001	0.875	0.384
		0.59	0.000	0.357	0.116	0.000	0.593	0.282	0.001	0.754	0.469	0.006	0.848	0.629
	0.5	0	0.000	0.500	0.031	0.000	0.785	0.117	0.000	0.916	0.269	0.000	0.973	0.466
		0.14	0.001	0.512	0.046	0.000	0.782	0.148	0.001	0.914	0.312	0.003	0.966	0.503
		0.39	0.002	0.528	0.103	0.005	0.780	0.272	0.011	0.888	0.470	0.032	0.942	0.618
		0.59	0.008	0.557	0.223	0.018	0.771	0.467	0.044	0.868	0.625	0.091	0.917	0.724
1	0	0	0.731	0.814	0.000	0.895	0.930	0.000	0.955	0.970	0.000	0.983	0.989	0.000
		0.14	0.694	0.760	0.000	0.858	0.885	0.000	0.922	0.934	0.000	0.956	0.960	0.000
		0.39	0.495	0.528	0.000	0.684	0.695	0.000	0.774	0.778	0.001	0.832	0.833	0.013
		0.59	0.278	0.308	0.000	0.488	0.489	0.002	0.600	0.601	0.017	0.679	0.679	0.081
	0.5	0	0.809	0.870	0.000	0.921	0.947	0.000	0.965	0.976	0.002	0.987	0.992	0.031
		0.14	0.790	0.843	0.000	0.899	0.925	0.001	0.943	0.958	0.007	0.968	0.975	0.062
		0.39	0.646	0.712	0.001	0.774	0.817	0.006	0.836	0.868	0.046	0.877	0.902	0.159
		0.59	0.470	0.557	0.005	0.621	0.685	0.041	0.700	0.754	0.146	0.757	0.803	0.263

*CFI_t values that were above the adjusted cut-off for close fit are bolded and underlined. The adjusted CFI cut-off values for N = 50 were: 0.77 – excellent; 0.66 – close; 0.59 – fair; 0.54 mediocre; <0.54 – poor. The adjusted CFI cut-off values for N = 100 were: 0.88 – excellent; 0.79 – close; 0.73 – fair; 0.69 mediocre; <0.69 – poor. The adjusted CFI cut-off values for N = 200 were: 0.94 – excellent; 0.86 – close; 0.80 – fair; 0.77 mediocre; <0.77 – poor. The adjusted CFI cut-off values for N = 500 were: 0.97 – excellent; 0.90 – close; 0.86 – fair; 0.83 mediocre; <0.83 – poor*.

#### Residualized Change Score Model

The CFI values for the residualized change score model were greater than the conventional cut-off of close fit except when stability = 0.30, baseline correlation = 0, and *b*_*y*2*m*2_ path = 0.59. The CFI values were also below the conventional cut-off of close fit when stability = 1.00 and the *b*_*y*2*m*2_ path was greater than or equal to 0.39. In general, the CFI values were larger in magnitude when the baseline correlation = 0.50 (see Table 1).

For *N* = 50, the CFI_t values for the residualized change score model were only above the adjusted cut-off of close-fit when stability = 1.00, baseline correlation = 0, and the *b*_*y*2*m*2_ path was less than or equal to 0.14 and when stability = 1.00, baseline correlation = 0.50, and the *b*_*y*2*m*2_ path was less than or equal to 0.39. For *N* = 100, the CFI_t values were greater than the adjusted cut-off of close fit for the same conditions as *N* = 50 with the addition of stability = 0, baseline correlation = 0.50, and the *b*_*y*2*m*2_ path = 0.59. For *N* = 200 and 500, the CFI_t values were greater than the adjusted cut-off of close fit when the baseline correlation = 0.50 except when stability = 1.00 and the *b*_*y*2*m*2_ path =0.59. In general, there were more conditions for which the CFI_t values were greater than the adjusted cut-off of close-fit as sample size increased and when the baseline correlation = 0.50 (see Table 2).

#### Cross-Sectional Model

The magnitude of the CFI values were above the conventional level of close fit when stability = 0. The only exception was when the baseline correlation = 0 and the *b*_*y*2*m*2_ path was less than or equal to 0.14 (see Table 1). For *N* = 50, there were no conditions for which the CFI_t values for the cross-sectional model were above the adjusted cut-off of close fit. For *N* = 100, the only condition for which the CFI_t value was greater than the adjusted level of close fit was when stability = 0, baseline correlation = 0.50, and the *b*_*y*2*m*2_ path = 0.59. For *N* =200, the only conditions for which the CFI_t values were above the adjusted cut-off of close fit was when stability =0, baseline correlation = 0, and the *b*_*y*2*m*2_ path = 0.59 and when stability = 0, baseline correlation = 0.50, and the *b*_*y*2*m*2_ path was greater than or equal to 0.39. For *N* = 500, CFI_t values were above the adjusted cut-off when stability = 0 and baseline correlation = 0.50. The CFI_t values were also above the adjusted cut-off level of close fit when stability = 0, baseline correlation = 0, and when the *b*_*y*2*m*2_ path was greater than or equal to 0.39 (see Table 2).

### RMSEA and *T*-size RMSEA Results

Because the RMSEA and *T*-size RMSEA (RMSEA_t) results were below the conventional cut-offs values when either or both the cross-lagged paths were greater than 0, the results were reported for the condition *M*_2_ cross-lag = 0 and *Y*_2_ cross-lag = 0. Both the RMSEA and RMSEA_t values were compared to their respective cut-off values. For the RMSEA values, the conventional cut-offs are: 0.01 – excellent; 0.05 – close; 0.08 – fair; 0.10 mediocre; >0.10 – poor. The adjusted RMSEA cut-off values are estimated based on the observed sample size and model degrees of freedom. Because the models tested contain the same degrees of freedom, the adjusted RMSEA cut-off values varied as only as a function of sample size therefore the RMSEA_t results were reported for each sample size. The adjusted RMSEA cut-off values for *N* = 50 were: 0.22 – excellent; 0.23 – close; 0.24 – fair; 0.25 mediocre; >0.25 – poor. The adjusted RMSEA cut-off values for *N* = 100 were: 0.15 – excellent; 0.16 – close; 0.19 – fair; 0.20 mediocre; >0.20 – poor. The adjusted RMSEA cut-off values for *N* = 200 were: 0.11 – excellent; 0.13 – close; 0.15 – fair; 0.17 mediocre; >0.17 – poor. The adjusted RMSEA cut-off values for *N* = 500 were: 0.07 – excellent; 0.10 – close; 0.12 – fair; 0.14 mediocre; >0.14 – poor.

#### Difference Score Model

Table 3 displays the RMSEA results tabled as a function of the stability, *b*_*y*2*m*2_ path, and baseline correlation for all models. The only conditions that resulted in RMSEA values below the conventional cut-off for close-fit was when stability = 1.00 and the *b*_*y*2*m*2_ path = 0 (see Table 3).

**Table 3:** RMSEA values for the difference score model (Diff), residualized change score model (Res), and cross-sectional model (Cross) for *M*_2_cross-lag = 0 and *Y*_2_ cross-lag = 0.

		**Base. Corr**.					
		**0**			**0.5**		
		**Diff**	**Res**	**Cross**	**Diff**	**Res**	**Cross**
Stability	b_*y*2*m*2_ path						
0	0	0.588	0.012	0.029	0.604	0.010	0.029
	0.14	0.589	0.012	0.029	0.598	0.011	0.029
	0.39	0.597	0.016	0.029	0.589	0.014	0.029
	0.59	0.614	0.025	0.029	0.589	0.022	0.029
0.3	0	0.448	0.011	0.198	0.468	0.011	0.211
	0.14	0.450	0.014	0.199	0.457	0.014	0.211
	0.39	0.469	0.045	0.198	0.448	0.038	0.212
	0.59	0.505	0.096	0.198	0.460	0.079	0.211
1	0	0.029	0.010	0.588	0.029	0.010	0.604
	0.14	0.065	0.046	0.588	0.065	0.038	0.604
	0.39	0.197	0.187	0.588	0.198	0.160	0.605
	0.59	0.326	0.321	0.588	0.326	0.282	0.604

*RMSEA values that were above the conventional cut-off for close fit are bolded and underlined. The conventional cut-offs are: 0.01 – excellent; 0.05 – close; 0.08 – fair; 0.10 mediocre; >0.10 - poor*.

For *N* = 50, the RMSEA_t values for the difference score model were only below the adjusted cut-off for close fit when stability = 1.00 and when the *b*_*y*2*m*2_ path was less than or equal to 0.14 which was true for both values of the baseline correlation. For *N* = 100 – 500, the RMSEA_t values were only below the adjusted cut-off for close fit when stability = 1.00 and when the *b*_*y*2*m*2_ path = 0 which was true for both values of the baseline correlation (see Table 4).

**Table 4:** *T*-size RMSEA values (RMSEA_t) for the difference score model (Diff), residualized change score model (Res), and cross-sectional model (Cross) for *M*_2_cross-lag = 0 and *Y*_2_ cross-lag = 0.

			**Sample Size**											
			**50**			**100**			**200**			**500**		
			**Diff**	**Res**	**Cross**	**Diff**	**Res**	**Cross**	**Diff**	**Res**	**Cross**	**Diff**	**Res**	**Cross**
Stability	Base. Corr.	b_*y*2*m*2_ path												
0	0	0	0.710	0.168	0.205	0.673	0.111	0.143	0.647	0.081	0.100	0.626	0.048	0.062
		0.14	0.710	0.165	0.207	0.675	0.114	0.142	0.649	0.078	0.099	0.627	0.050	0.062
		0.39	0.718	0.179	0.206	0.682	0.120	0.141	0.657	0.086	0.098	0.636	0.054	0.062
		0.59	0.735	0.197	0.206	0.699	0.135	0.141	0.674	0.094	0.099	0.652	0.059	0.062
	0.5	0	0.726	0.160	0.205	0.689	0.106	0.141	0.664	0.078	0.099	0.642	0.048	0.062
		0.14	0.720	0.159	0.205	0.683	0.108	0.141	0.657	0.082	0.100	0.635	0.048	0.062
		0.39	0.710	0.168	0.204	0.674	0.117	0.141	0.649	0.081	0.099	0.627	0.053	0.063
		0.59	0.710	0.192	0.205	0.674	0.131	0.142	0.649	0.090	0.099	0.627	0.056	0.062
0.3	0	0	0.571	0.163	0.332	0.533	0.110	0.291	0.508	0.078	0.265	0.487	0.048	0.242
		0.14	0.573	0.163	0.333	0.536	0.117	0.292	0.510	0.085	0.264	0.489	0.059	0.242
		0.39	0.593	0.197	0.333	0.554	0.151	0.291	0.530	0.122	0.264	0.507	0.100	0.242
		0.59	0.628	0.243	0.333	0.591	0.197	0.291	0.565	0.170	0.264	0.543	0.149	0.241
	0.5	0	0.592	0.161	0.343	0.554	0.109	0.302	0.528	0.077	0.276	0.507	0.049	0.254
		0.14	0.581	0.166	0.343	0.544	0.116	0.302	0.516	0.083	0.277	0.495	0.057	0.254
		0.39	0.571	0.191	0.345	0.534	0.142	0.303	0.508	0.113	0.277	0.486	0.091	0.255
		0.59	0.584	0.228	0.345	0.546	0.182	0.302	0.520	0.153	0.276	0.498	0.134	0.255
1	0	0	0.205	0.161	0.710	0.141	0.110	0.673	0.098	0.077	0.647	0.063	0.048	0.626
		0.14	0.227	0.191	0.710	0.171	0.149	0.672	0.137	0.123	0.648	0.111	0.105	0.625
		0.39	0.330	0.312	0.710	0.290	0.280	0.673	0.263	0.259	0.647	0.241	0.239	0.626
		0.59	0.452	0.443	0.710	0.413	0.409	0.674	0.388	0.385	0.647	0.366	0.365	0.626
	0.5	0	0.204	0.159	0.726	0.142	0.111	0.690	0.099	0.078	0.664	0.062	0.048	0.642
		0.14	0.225	0.183	0.726	0.171	0.141	0.689	0.137	0.114	0.664	0.111	0.095	0.642
		0.39	0.333	0.288	0.728	0.291	0.255	0.690	0.264	0.233	0.664	0.241	0.214	0.642
		0.59	0.452	0.402	0.726	0.414	0.371	0.688	0.389	0.348	0.665	0.365	0.328	0.642

*RMSEA_t values that were above the conventional cut-off for close fit are bolded and underlined. The adjusted RMSEA cut-off values for N = 50 were: 0.22 – excellent; 0.23 – close; 0.24 – fair; 0.25 mediocre; >0.25 – poor. The adjusted RMSEA cut-off values for N = 100 were: 0.15 – excellent; 0.16 – close; 0.19 – fair; 0.20 mediocre; >0.20 – poor. The adjusted RMSEA cut-off values for N = 200 were: 0.11 – excellent; 0.13 – close; 0.15 – fair; 0.17 mediocre; >0.17 – poor. The adjusted RMSEA cut-off values for N = 500 were: 0.07 – excellent; 0.10 – close; 0.12 – fair; 0.14 mediocre; >0.14 – poor*.

#### Residualized Change Score Model

When stability = 0.00, the RMSEA was below the conventional cut-off value for close fit for all values of the *b*_*y*2*m*2_ path and for both values of the baseline correlation. When stability = 0.30, the RMSEA was below the conventional cut-off value for close fit when the *b*_*y*2*m*2_ path was less than or equal to 0.39 for both values of the baseline correlation. When stability = 1.00, the RMSEA was below the conventional cut-off value for close fit when the *b*_*y*2*m*2_ path was less than or equal to 0.14 for both values of the baseline correlation (see Table 3).

For *N* = 50, the RMSEA_t values for the residualized change score model were below the adjusted cut-off value for close fit when stability was less than or equal to 0.30 except when the baseline correlation = 0.50 and the *b*_*y*2*m*2_ path = 0.59. When stability =1.00, the RMSEA_t values were below the adjusted cut-off value for close fit when the *b*_*y*2*m*2_ path was less than or equal to 0.14 across both values of the baseline correlation. For *N* = 100 – 500, the RMSEA_t values for the residualized change score model were below the adjusted cut-off value for close fit when stability = 0. When stability = 0.30, the RMSEA_t values were below the adjusted cut-off value for close-fit when the *b*_*y*2*m*2_ path was less than or equal to 0.39 across both values of the baseline correlation. When stability =1.00, the RMSEA_t values were below the adjusted cut-off value for close fit when the *b*_*y*2*m*2_ path was less than or equal to 0.14 across both values of the baseline correlation (see Table 4).

#### Cross-Sectional Model

The only conditions that resulted in RMSEA values below the conventional cut-off for close-fit was when stability = 0.00 (see Table 3). For all sample sizes, the RMSEA_t values for the cross-sectional model were only below the adjusted cut-off value for close fit when stability = 0.00 (see Table 4).

### Summary of Simulation Results

A consistent pattern emerged for the model fit for the difference score and cross-sectional models across the χ^2^ test, CFI, *T*-size CFI, RMSEA, *T*-size RMSEA, and the additional fit indices reported in the **Supplemental Materials**. For the difference score model, Type 1 error rates for the χ^2^ test were within the robustness interval, CFI and *T*-size CFI values were above their respective cut-off values for close-fit, and RMSEA and *T*-size RMSEA values were below their respective cut-off values for close-fit when stability = 1.00, the cross-lagged effects = 0.00, and the *b*_*y*2*m*2_ path was less than or equal to 0.14. For the residualized change score model, when both cross-lagged paths = 0, the Type 1 error rates of the χ^2^ test increased as both stability and the *b*_*y*2*m*2_ path increased in magnitude. The CFI values were above the conventional cut-off for close-fit and the RMSEA values were below the conventional cut-off for close-fit primarily when stability was less than or equal to 0.30. As stability increased, the fit according to the CFI and RMSEA got worse as the magnitude of the *b*_*y*2*m*2_ path increased. A similar pattern emerged for the *T*-size CFI and the *T*-size RMSEA values for the residualized change score model but only for sample sizes *N* = 200 and *N* = 500. For the cross-sectional model, Type 1 error rates for the χ^2^ test were within the robustness interval, CFI and *T*-size CFI values were above their respective cut-off values for close-fit, and RMSEA and *T*-size RMSEA values were below their respective cut-off values for close-fit when the stability and cross-lagged effects were equal = 0.00.

## Empirical Example

**Figure 2:**
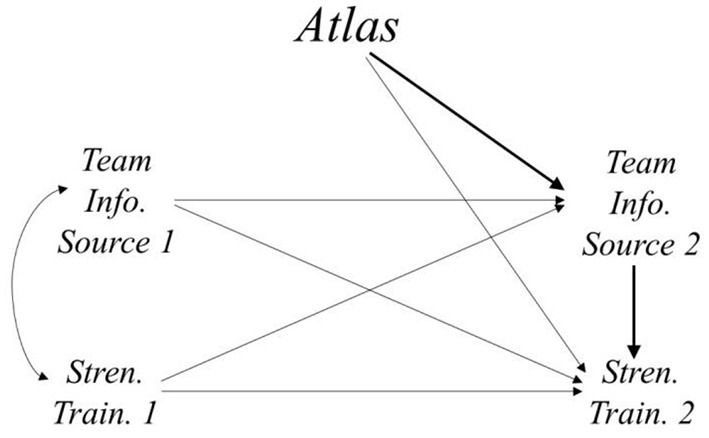
Diagram of the model described in the empirical example. The bold path from Atlas to Stren. Train. 2 through Team Info. Source 2 indicates the mediated effect of the ATLAS program on strength training self-efficacy at posttest through its effect on team as an information source at posttest.

Data from the Athletes Training and Learning to Avoid Steroids (ATLAS; Goldberg et al., 1996) program were used to demonstrate the model fit for the two-wave mediation models. MacKinnon et al. (2001) evaluated the mediating mechanisms of 12 mediators of the ATLAS program on three outcomes. In this example, the model tested students' perception of their high school football team as an information source at posttest as the mediating variable of the ATLAS program on strength training self-efficacy at posttest. The variables included pretest measures of the mediator, perception of team as information source at pretest (*M*_1_), which included items such as “Being on the football team has improved my health,” and the outcome, strength training self-efficacy at pretest (*Y*_1_), which included items such as “I know how to train with weights to become stronger.” Both the mediator and the outcome were measured immediately after the ATLAS program was administered (i.e., units randomly assigned to experimental conditions) and constitute the posttest measures of these variables, respectively (*M*_2_ and *Y*_2_) (see Figure 2).

There were 1,144 observations used in this example after listwise deletion of the original 1,506 observations. The purpose of the empirical example is to demonstrate that the models produce identical estimates of the mediated effect across the regression and LCS approaches but that the LCS approach has the added benefit of providing model fit information. Therefore, the models were estimated with both linear regression using the lm function in R and the LCS specification using the R package lavaan (Rosseel, 2012). Asymmetric distribution of the product confidence intervals were computed for the mediated effect estimates (MacKinnon et al., 2002; Preacher and Hayes, 2008) using the R package RMediation (Tofighi and MacKinnon, 2011). The equivalence tests were computed using the R function provided in Yuan et al. (2016) (syntax for a simulated dataset is provided in the Supplementary Materials).

The mediated effect estimate and 95% distribution of the product asymmetric confidence intervals (95% C.I.) for all models estimated with regression and the LCS specification were identical. That is, for the ANCOVA model the mediated effect estimate was 0.237 with 95% confidence interval equal to [0.181, 0.297] under both modeling strategies. The mediated effect estimate and 95% C.I. for the difference score model was 0.181, 95% C.I. [0.129, 0.239] and was identical under both modeling strategies. The mediated effect estimate and 95% C.I. for the residualized change score model was 0.222, 95% C.I. [0.168, 0.281] and was identical under both modeling strategies. Finally, the mediated effect estimate and 95% C.I. for the cross-sectional model was 0.254, 95% C.I. [0.188, 0.323] and was identical under both modeling strategies. In summary, linear regression and the LCS specification resulted in identical mediated effect estimates for the respective models.

The χ^2^ tests for the difference score model, residualized change score model, and cross-sectional models were all statistically significant. Therefore, the test provided evidence that the constraints implied by the difference score, residualized change score, and cross-sectional models did not fit the observed data well (see Table 5). The *T*-size RMSEA for the difference score model was 0.456. This implies we are 95% confident that the misspecification as measured by the RMSEA was not >0.456. Using the new cut-off values, the model had poor fit because the *T*-size RMSEA was greater than the adjusted poor fit cut-off value of 0.128. The *T*-size CFI for the difference score model was 0.000. This implies we are 95% confident that the population CFI is >0.000. Using the new cut-off values, our model had poor fit as measured by the CFI. The *T*-size RMSEA for the residualized change score model was 0.113. This implies we are 95% confident that the misspecification as measured by the RMSEA was not >0.113. Using the new cut-off values, the model had between fair and mediocre fit. Using the standard RMSEA value (0.087) and the standard cut-off values, the model fit would be considered between fair and mediocre as well. The *T*-size CFI for the difference score model was 0.930. This implies we are 95% confident that the population CFI is >0.930. Using the new cut-off values, the model fit was between excellent and close as measured by the CFI. Using the standard CFI (0.986) and the standard cut-off values, the model fit was between excellent and close. The *T*-size RMSEA for the cross-sectional model was 0.314. This implies we are 95% confident that the misspecification as measured by the RMSEA was no greater than 0.314. Using the new cut-off values, the model fit was poor. The *T*-size CFI for the cross-sectional model was 0.488. This implies we are 95% confident that the population CFI was >0.488. Using the new cut-off values, our model fit was poor.

**Table 5:** Model fit information for LCS specification of two-wave mediation models applied to empirical example.

**Model**	**Baseline χ^2^ (*df*)**	**Model χ^2^ (*df*)**	**RMSEA**	**RMSEA T-size**	**New RMSEA cut-off**	**CFI**	**CFI T-size**	**New CFI cut-offs**
ANCOVA	1039.801 (10)	0 (0)	0	NA	NA	1	NA	NA
Diff	1039.801 (10)	855.026 (4)	0.431	0.456	0.047; 0.079; 0.108; 0.128;	0.174	0.000	0.857; 0.882; 0.921; 0.977
Res	1039.801 (10)	38.769 (4)	0.087	0.113	0.047; 0.079; 0.108; 0.128;	0.986	0.930	0.857; 0.882; 0.921; 0.977
Cross	1039.801 (10)	387.234 (4)	0.289	0.314	0.047; 0.079; 0.108; 0.128;	0.628	0.488	0.857; 0.882; 0.921; 0.977

In summary, we demonstrated with the empirical example that researchers can estimate the mediated effect under the ANCOVA, difference score, residualized change score, and cross-sectional models using linear regression and the LCS specification which provide identical mediated effect estimates for the respective models. The added benefit of the LCS specification is that it makes explicit the model constraints assumed by the difference score, residualized change score, and cross-sectional models and provides evidence of the fit of these models *via* model fit indices. We tested the model constraints for the difference score, residualized change score, and cross-sectional models in our example and we found evidence that the non-ANCOVA models did not fit the data as well as the ANCOVA model although the residualized change score model did not fit poorly. Overall, we would proceed by selecting the ANCOVA model. However, it is possible the model misfit in the empirical example was caused by other sources of model misspecification (more on this in the discussion section). Overall, the χ^2^ tests, CFI, *T*-size CFI, RMSEA, and *T*-size RMSEA provided the same general conclusion that the difference score, residualized change score, and cross-sectional models did not fit the data well. The *T*-size model fit indices of RMSEA and CFI provide a different interpretation than the standard RMSEA and CFI but comparing these *T*-size fit indices to the adjusted cut-off values provided the same conclusion of model fit for each model.

## Discussion

This paper extended previous work using the LCS specification to estimate two-wave mediation models by testing the performance of model fit statistics when evaluating which of the two-wave mediation models best describe the observed data. The goals of this paper were to describe how researchers can use the LCS specification to fit the two-wave mediation models and demonstrate conditions under which goodness-of-fit indexes including newly proposed *T*-size fit indices perform well. The LCS specification of the two-wave mediation models provides an advantage over the regression approach because the LCS specification allows researchers to test the implications of the model constraints imposed by each of the nested two-wave mediation models *via* model fit indices. This is an important strength over the regression approach which implicitly assumes the nested two-wave mediation models fit the data equally well as the ANCOVA model.

Overall, the χ^2^ tests for the models (difference score, residualized change score, and cross-sectional) had Type 1 error rates within the robustness interval for a limited range of conditions corresponding to the strict assumptions each model makes regarding the magnitude of the stabilities and cross-lagged paths. Subsequently, the χ^2^ tests for the models resulted in high statistical power to reject the null hypothesis that the nested and full model (e.g., the difference score and ANCOVA model) fit the data equally well. The CFI and RMSEA fit indexes generally provided similar information regarding the fit of the difference score, residualized change score, and cross-sectional models. The *T*-size counterparts of the CFI and RMSEA provided similar fit conclusions as the CFI and RMSEA but these conclusions were dependent on sample size.

Although the *T*-size CFI and *T*-size RMSEA resulted in similar fit conclusions as the CFI and RMSEA, respectively, the *T*-size measures have a different interpretation than the standard CFI and RMSEA values. For example, at α = 0.05, the *T*-size CFI is interpreted as “we are 95% confident that the population CFI is above *X*” and the *T*-size RMSEA is interpreted as “we are 95% confident that the misspecification is *X-*units as measured by the RMSEA” (Marcoulides and Yuan, 2017). Therefore, the *T*-size measures provide researchers with information regarding the probability that the size of the model misspecification is within a certain tolerable size. This is useful additional information that researchers may want to consider when fitting two-wave longitudinal mediation models. The *T*-size measures can easily be calculated using an R function created by Yuan et al. (2016).

An interesting result was that the magnitude of the mediator-outcome relation at posttest (*b*_*y*2*m*2_ in Figure 1A) negatively affected the fit of the difference score and residualized change score models. Upon inspection of the residual covariance matrices for some of these conditions, it appeared that the covariance between the pretest mediator and latent change score for the outcome was equal to zero. This covariance term is non-zero in the population but is assumed to be equal to zero *via* path tracing rules when using the LCS specification (see Supplementary Material for more details). Because of the complexity of the relations between the mediator-outcome relation at posttest across the different models, further research is needed to fully understand the factors involved in affecting the model fit as the mediator-outcome relation increases in magnitude. Further, the effect the magnitude of the mediator-outcome relation at posttest had on the model fit may be explained by conceptual differences in how the time interval between measurement occasions is encoded by the models.

### Conceptual Differences Between the Models of Change

In applied settings, it is important to consider the conceptual differences between the models of change. The ANCOVA model is a conditional model of change and the difference score model is an unconditional model of change. Consequently, the ANCOVA model and difference score model make different assumptions about *regression to the mean*. Absent a treatment effect, the ANCOVA model assumes regression to the mean will occur with the most extreme values at pretest becoming less extreme at posttest (i.e., regressing to the mean at posttest) while the difference score model assumes individual differences at pretest will maintain through posttest (Cronbach and Furby, 1970; Dwyer, 1983; Rogosa, 1988; Campbell and Kenny, 1999). In other words, the ANCOVA model assumes that individuals with the most extreme values on the mediator and the outcome variables at pretest will tend toward the mean of the mediator and the outcome variables at posttest, respectively. The difference score model assumes individual differences on the mediator and the outcome variables at pretest will remain the same at posttest which is encoded in the assumption that the stabilities of the mediator and the outcome variables are equal to one. The residualized change score model provides a middle ground between the ANCOVA and difference score models by computing a change variable that is the difference between the observed posttest values of the mediator and outcome and predicted posttest values (using the pretest values) of the mediator and outcome, respectively. The cross-sectional model is not a model for change since it makes no adjustment for pretest values either conditionally or unconditionally.

Another important theoretical consideration is that of the time-interval between measurement occasions. The time-interval between the measurements of the treatment, mediator, and the outcome will play a role in the effect size of those relations (Gollob and Reichardt, 1991; Collins and Graham, 2002; Cole and Maxwell, 2003; Reichardt, 2011). This holds for the stabilities of the variables, the cross-sectional relations, and the cross-lagged effects. For example, the magnitude of the effect of the mediator on the outcome will be different if the mediator and outcome are measured 30 min apart vs. 3 months apart. What is considered a short time interval for change to occur in one variable (e.g., *M*) may not be a short time interval for change to occur in another variable (e.g., *Y*) and the time-interval that produces the largest effect sizes does not necessarily mean this is the “correct” time-interval. It is preferred to have measured *X, M*, and *Y* over a time-interval that is believed to represent the underlying theoretical process of change. In other words, simply measuring the mediator before the outcome does not mean the mediator construct precedes the outcome construct.

### Implications and Recommendations

When researchers have questions that are explicitly about relating change in the mediator over a period of time to the change in the outcome over a period of time, the difference score and residualized change score model may seem appealing but each model requires model constraints that can be explicitly tested with the LCS specification of the two-wave mediation model. The LCS specification of the two-wave mediation model makes it clear that the difference score and residualized change score models make strict assumptions regarding the stability of the mediator and outcome and the cross-lagged paths. In this case, researchers may want to take this information into consideration when choosing which two-wave mediation model will best represent their observed data.

Overall, it is recommended that researchers consider the model of change that describes the phenomenon of under investigation and are encouraged to report model fit information such as the CFI and RMSEA values and the *T*-size CFI and *T*-size RMSEA to supplement the results of the χ^2^ test. These recommendations are meant to provide researchers with an additional tool when evaluating models of change for the two-wave mediation model. If researchers are planning on fitting two-wave mediation models, they may want to rely on the theory of change that best represents the phenomena under investigation and supplement this model choice with statistical evidence *via* the fit indices mentioned above.

### Limitations and Future Directions

The two-wave mediation models described in this manuscript assume the absence of treatment-by-mediator interactions. The treatment-by-mediator interaction is an important component to mediation modeling in the potential outcomes framework for causal inference (Vanderweele and Vansteelandt, 2009; Valeri and Vanderweele, 2013; Mackinnon et al., 2020). The presence of a treatment-by-mediator interaction implies the mediated effects may differ in magnitude across the control and treatment groups and the direct effects may differ in magnitude across levels of the mediator variable. It is unclear how the presence of treatment-by-mediator interactions may impact the performance of the two-wave mediation models described in this manuscript or how these interactions may manifest over the repeated measurements (e.g., baseline-by-treatment interactions; MacKinnon, 2008, Ch. 8; Morgan-Lopez and MacKinnon, 2006).

It was assumed in this manuscript that the mediator variable construct had identical measurement properties across the control and treatment groups and across time. In other words, it was assumed there was measurement invariance. Measurement invariance in the mediation model has become an interesting area of research because the causal conclusions of mediation analysis rests on measuring the same construct for each group at each time point. Recent work has begun to incorporate concepts and statistical tests of measurement invariance into the mediation model in general and the two-wave mediation model in particular (Olivera-Aguilar et al., 2018; Georgeson et al., 2021)^2^ and more generally to investigate properties of mediation models under varying psychometric properties (Gonzalez and MacKinnon, 2016, 2020). More work is needed to determine how psychometric properties of the mediator and outcome may affect statistical mediation conclusions. In general, it is recommended that researchers consider the measurement theory and use latent variables whenever possible.

It was assumed that model misfit as characterized in this study was caused by misspecification of the models of change estimated in the sample compared to their population counterpart. Model misspecification could occur for a variety of reasons including the presence of unmeasured confounders (Holland, 1988; Imai et al., 2010), measurement error (Fritz et al., 2016), lack of measurement invariance across groups or over time (Olivera-Aguilar et al., 2018; Georgeson et al., 2021)^2^, and misspecified non-linear or non-additive effects like treatment-by-mediator interactions (Vanderweele and Vansteelandt, 2009; Valeri and Vanderweele, 2013; Mackinnon et al., 2020). Therefore, researchers may also consider these other potential sources of model misspecification when comparing models of change.

## Conclusion

Overall, the LCS approach to two-wave mediation models provides a strong advantage over the traditional regression approach for these models because the LCS specification can be used to assess the adequacy of the assumed model constraints of the difference score, residualized change score, and cross-sectional model. Our research demonstrates that traditional and newly-proposed model fit indices perform well in distinguishing models of change. Specifically, in addition to choosing the model of change that best describes the phenomenon under investigation—including an evaluation of the plausibility of the constraints implied by the difference score, residualized change score, and cross-sectional models—the model fit indices were generally able to provide evidence in support of a more parsimonious models when the model the constraints of the parsimonious models held in the population. We encourage researchers to use the LCS specification to assess if their model of change is adequate for their data.

## Data Availability Statement

The data analyzed in this study is subject to the following licenses/restrictions: the PI of the original dataset has not made the dataset publicly available. Requests to access these datasets should be directed to mvalente@fiu.edu.

## Author Contributions

MV came up with the idea, ran the simulation, and wrote the manuscript. AG and OG wrote and edited the manuscript. All authors contributed to the article and approved the submitted version.

## Conflict of Interest

The authors declare that the research was conducted in the absence of any commercial or financial relationships that could be construed as a potential conflict of interest.

## Publisher's Note

All claims expressed in this article are solely those of the authors and do not necessarily represent those of their affiliated organizations, or those of the publisher, the editors and the reviewers. Any product that may be evaluated in this article, or claim that may be made by its manufacturer, is not guaranteed or endorsed by the publisher.

## Footnotes

^1^It is assumed that the mediator at posttest occurs prior to the outcome at posttest. Research designs with multiple posttest follow-up waves may be preferred when establishing temporal precedence of the mediator – outcome relation (Gollob and Reichardt, 1991; Cole and Maxwell, 2003; MacKinnon, 2008). However, in this paper we focus on the traditional pretest-posttest control group design. To assume that the mediated effect in the model has a causal interpretation, we assume that there are no unmeasured confounders of treatment-mediator relation conditional on the pretest measures, no unmeasured confounders of the mediator-outcome relation conditional on treatment and the pretest measures, no unmeasured confounders of the treatment-outcome relation conditional on the pretest measures, and no post-treatment confounders of the mediator-outcome relation affected by treatment conditional on the pretest measures (MacKinnon, 2008; Vanderweele and Vansteelandt, 2009; Valente and MacKinnon, 2017; Valente et al., 2020).

^2^Georgeson, A. R., Valente, M. J., and Gonzalez, O. (2020). The effect of partial invariance on mediation in a two-wave mediation model for interventions.
