# Supplementary material for: Clarifying the Implicit Assumptions of Two-Wave Mediation Models via the Latent Change Score Specification: An Evaluation of Model Fit Indices
Source: Front Psychol. 2021 Sep 6;12:709198. doi: 10.3389/fpsyg.2021.709198 (PMC8450329; doi:10.3389/fpsyg.2021.709198)
Supplement: Supplementary file 1 [file Data_Sheet_1.docx]

Supplemental materials containing additional results for model comparisons and covariance algebra for “Clarifying the Implicit Assumptions of Two-Wave Mediation Models Via the Latent Change Score Specification: An Evaluation of Model Fit Indices”, *Frontiers in Psychology*.

**Algebra Comparing Difference score and Residualized Change Score Models to ANCOVA**

An investigation of the true covariances for the difference score, residualized chance score, and ANCOVA models reveals both the difference score and residualized change score models likely underestimate the covariance between the mediator at pretest and outcome at posttest by a factor of $b_{\Delta}\sigma_{M1M2}$ for the difference score model and $b_{Res\Delta}\sigma_{M1M2}$ for the residualized change score model. Using path tracing rules on the LCS path models displayed in Figure 1B and 1C in the main manuscript, it is implied that the difference score and residualized change score models constrain $Cov\left( M_{1},\Delta Y \right)=0$.

The full covariance between the pretest mediator and the posttest outcome for the ANCOVA model is equal to:

| $Cov\left( M_{1}, Y_{2} \right)=s_{Y2Y1}\sigma_{M1Y1}+b_{Y2M1}\sigma_{M1}^{2}+b_{Y2M2}\sigma_{M1M2}$ | (1) |
| --- | --- |

For the difference score model and assuming cross-lags = 0 and stability = 1.00 and baseline correlation = 0, the population $Cov\left( M_{1},{\Delta Y}_{2} \right)$for the difference score model is equal to:

| $Cov[M_{1}, \Delta Y)] = b_{\Delta}\sigma_{M1M2}$ | (2) |
| --- | --- |

For the residualized change score model and assuming cross-lags = 0 and stability = 1.00 and baseline correlation = 0, the population $Cov\left( M_{1},Res\Delta Y \right)$for the residualized change score model is equal to:

| $Cov[M_{1}, Res\Delta Y)] = b_{Res\Delta}\sigma_{M1M2}$ | (3) |
| --- | --- |

Therefore, the population values of the covariance between the pretest mediator and the latent change score for the difference score and residualized change score models are non-zero in the population but are assumed zero in the LCS specification of the models.

**Table of All Significant Predictors of LR test, CFI, *T*-size CFI, RMSEA, *T*-size RMSEA, SRMR, AIC, and BIC**

Table S1 displays the significant predictors of the LR test for the difference score, residualized change score, and cross-sectional models that had semi-partial eta-squared values of 0.005 (within rounding) or greater.

| Table S1 | | | |
| --- | --- | --- | --- |
| *Semi-partial η^2^ for Predictors of LR test* | | | |
| Effect Type/Variables | Difference Score | Residualized Change Score | Cross-sectional |
| **Main Effects** |  |  |  |
| Stability | 0.0692 | 0.0165 | 0.0910 |
| *b* path | 0.0293 | 0.0185 | N/A |
| *M_2_* lag | 0.0344 | 0.2125 | 0.0888 |
| *Y_2_* lag | 0.0378 | 0.2429 | 0.0972 |
|  |  |  |  |
| **Two-way interactions** |  |  |  |
| *M_2_* lag X *Y_2_* lag | 0.0343 | 0.2101 | 0.0885 |
| Stability X *M_2_* lag | 0.0629 | 0.0151 | 0.0832 |
| Stability X *Y_2_* lag | 0.0691 | 0.0163 | 0.0907 |
| *b* path X Stability | 0.0536 | 0.0094 | N/A |
| *b* path X *M_2_* lag | 0.0268 | 0.0139 | N/A |
| *b* path X *Y_2_* lag | 0.0292 | 0.0181 | N/A |
|  |  |  |  |
|  |  |  |  |
| **Three-way interactions** |  |  |  |
| Stability X *M_2_* lag X *Y_2_* lag | 0.0628 | 0.0149 | 0.0829 |
| *b* path X Stability X *M_2_* lag | 0.0490 | 0.0088 | N/A |
| *b* path X Stability X *Y_2_* lag | 0.0535 | 0.0094 | N/A |
| *b* path X *M_2_* lag X *Y_2_* lag | 0.0267 | 0.0135 | N/A |
|  |  |  |  |
| **Four-Way interactions** |  |  |  |
| *b* path X Stability X *M_2_* lag X *Y_2_* lag | 0.0489 | 0.0087 | N/A |

Table S2 displays the significant predictors of the CFI values for the difference score, residualized change score, and cross-sectional models that had semi-partial eta-squared values of 0.005 (within rounding) or greater.

| Table S2 | | | |
| --- | --- | --- | --- |
| *Semi-partial η^2^ for Predictors of CFI* | | | |
| Effect Type/Variables | Difference Score | Residualized Change Score | Cross-sectional |
| **Main Effects** |  |  |  |
| Baseline correlation | 0.0450 | 0.1001 | 0.0554 |
| Stability | 0.7384 | 0.0588 | 0.1880 |
| *a* path | 0.0051 | 0.0199 | 0.0493 |
| *b* path | N/A | N/A | 0.1024 |
| *c’* path | N/A | N/A | 0.0120 |
| *M_2_* lag | N/A | 0.1216 | 0.0277 |
| *Y_2_* lag | 0.0069 | 0.4215 | 0.1339 |
|  |  |  |  |
| **Two-way interactions** |  |  |  |
| *M_2_* lag X *Y_2_* lag | N/A | 0.0374 | 0.0271 |
| Stability X *M_2_* lag | 0.0145 | 0.0127 | 0.0646 |
| Stability X *Y_2_* lag | 0.0422 | 0.0124 | 0.1459 |
| *a* path X Stability | N/A | 0.0065 | 0.0047 |
| *b* path X Stability | 0.0106 | 0.0265 | N/A |
| Baseline correlation X *M_2_* lag | 0.0080 | 0.0047 | N/A |
| Baseline correlation X *Y_2_* lag | 0.0053 | 0.0255 | N/A |
| Baseline correlation X Stability | N/A | 0.0063 | N/A |
| Baseline correlation X *a* path | N/A | 0.0047 | 0.0073 |
| Baseline correlation X *b* path | N/A | N/A | 0.0083 |
|  |  |  |  |
| **Three-way interactions** |  |  |  |
| Stability X *M_2_* lag X *Y_2_* lag | N/A | 0.0104 | 0.0102 |

Table S3 displays the significant predictors of the CFI_t values for the difference score, residualized change score, and cross-sectional models that had semi-partial eta-squared values of 0.005 (within rounding) or greater.

| Table S3 | | | |
| --- | --- | --- | --- |
| *Semi-partial η^2^ for Predictors of CFI_t* | | | |
| Effect Type/Variables | Difference Score | Residualized Change Score | Cross-sectional |
| **Main Effects** |  |  |  |
| Baseline correlation | 0.0185 | 0.0771 | 0.0274 |
| Stability | 0.5956 | 0.1064 | 0.0763 |
| *a* path | N/A | 0.0172 | 0.0374 |
| *b* path | 0.0045 | N/A | 0.0703 |
| *c’* path | N/A | N/A | 0.0070 |
| *M_2_* lag | N/A | 0.0976 | 0.0280 |
| *Y_2_* lag | 0.0456 | 0.2684 | 0.0932 |
| Nobs | 0.0281 | 0.0973 | 0.1270 |
|  |  |  |  |
| **Two-way interactions** |  |  |  |
| *M_2_* lag X *Y_2_* lag | N/A | 0.0611 | 0.0362 |
| Stability X *M_2_* lag | 0.0142 | 0.0046 | 0.0507 |
| Stability X *Y_2_* lag | 0.0943 | N/A | 0.0898 |
| Stability X Nobs | 0.0266 | N/A | 0.0098 |
| *Y_2_* lag X Nobs | N/A | N/A | 0.0086 |
| *b* path X Nobs | N/A | N/A | 0.0108 |
| *a* path X Stability | N/A | N/A | 0.0092 |
| *b* path X Stability | 0.0147 | 0.0264 | N/A |
| *a* path X *Y_2_* lag | N/A | N/A | 0.0050 |
| *b* path X *Y_2_* lag | N/A | N/A | 0.0093 |
| Baseline correlation X Stability | 0.0091 | N/A | N/A |
| Baseline correlation X Nobs | N/A | N/A | 0.0067 |
|  |  |  |  |
| **Three-way interactions** |  |  |  |
| Stability X *M_2_* lag X *Y_2_* lag | N/A | 0.0084 | 0.0193 |
| Stability X *M_2_* lag X Nobs | N/A | N/A | 0.0053 |
| Stability X *Y_2_* lag X Nobs | N/A | N/A | 0.0115 |
| Stability X *Y_2_* lag X *b* path | N/A | N/A | 0.0066 |

Table S4 displays the significant predictors of the RMSEA values for the difference score, residualized change score, and cross-sectional models that had semi-partial eta-squared values of 0.005 (within rounding) or greater.

| Table S4 | | | |
| --- | --- | --- | --- |
| *Semi-partial η^2^ for Predictors of RMSEA* | | | |
| Effect Type/Variables | Difference Score | Residualized Change Score | Cross-sectional |
| **Main Effects** |  |  |  |
| Baseline correlation | 0.0088 | N/A | 0.0084 |
| Stability | 0.3930 | 0.0388 | 0.5741 |
| *b* path | 0.0560 | 0.0532 | N/A |
| *M_2_* lag | 0.0117 | 0.2083 | 0.0670 |
| *Y_2_* lag | 0.1637 | 0.5362 | 0.1785 |
|  |  |  |  |
| **Two-way interactions** |  |  |  |
| *M_2_* lag X *Y_2_* lag | 0.0074 | 0.0436 | 0.0143 |
| Stability X *M_2_* lag | 0.0558 | N/A | 0.0292 |
| Stability X *Y_2_* lag | 0.1258 | N/A | N/A |
| *b* path X Stability | 0.0419 | 0.0282 | N/A |
| Baseline correlation X Stability | 0.0078 | N/A | N/A |
|  |  |  |  |
| **Three-way interactions** |  |  |  |
| Stability X *b* path X *Y_2_* lag | 0.0056 | N/A | N/A |

Table S5 displays the significant predictors of the RMSEA_t values for the difference score, residualized change score, and cross-sectional models that had semi-partial eta-squared values of 0.005 (within rounding) or greater.

| Table S5 | | | |
| --- | --- | --- | --- |
| *Semi-partial η^2^ for Predictors of RMSEA_t* | | | |
| Effect Type/Variables | Difference Score | Residualized Change Score | Cross-sectional |
| **Main Effects** |  |  |  |
| Baseline correlation | 0.0089 | N/A | 0.0086 |
| Stability | 0.3733 | 0.0412 | 0.5763 |
| *b* path | 0.0541 | 0.0579 | N/A |
| *M_2_* lag | 0.0106 | 0.2140 | 0.0643 |
| *Y_2_* lag | 0.1593 | 0.5562 | 0.1747 |
| Nobs | 0.0397 | 0.0278 | 0.0249 |
|  |  |  |  |
| **Two-way interactions** |  |  |  |
| *M_2_* lag X *Y_2_* lag | 0.0066 | 0.0423 | 0.0127 |
| Stability X *M_2_* lag | 0.0527 | N/A | 0.0272 |
| Stability X *Y_2_* lag | 0.1200 | N/A | 0.0600 |
| *b* path X Stability | 0.0395 | 0.0296 | N/A |
| Baseline correlation X Stability | 0.0078 | N/A | N/A |
|  |  |  |  |
| **Three-way interactions** |  |  |  |
| Stability X *b* path X *Y_2_* lag | 0.0047 | N/A | N/A |

Table S6 displays the significant predictors of the Standardized Root Mean Square Residual (SRMR) for the difference score, residualized change score, and cross-sectional models that had semi-partial eta-squared values of 0.005 (within rounding) or greater.

| Table S6 | | | |
| --- | --- | --- | --- |
| *Semi-partial η^2^ for Predictors of SRMR* | | | |
| Effect Type/Variables | Difference Score | Residualized Change Score | Cross-sectional |
| **Main Effects** |  |  |  |
| Baseline correlation | 0.0048 | N/A | N/A |
| Stability | 0.6063 | N/A | N/A |
| *a* path | 0.0123 | N/A | N/A |
| *b* path | 0.0130 | N/A | N/A |
| *M_2_* lag | 0.0244 | N/A | N/A |
| *Y_2_* lag | 0.0075 | N/A | N/A |
|  |  |  |  |
| **Two-way interactions** |  |  |  |
| Stability X *M_2_* lag | 0.0447 | N/A | N/A |
| Stability X *Y_2_* lag | 0.0439 | N/A | N/A |
| *a* path X Stability | 0.0052 | N/A | N/A |
| *b* path X Stability | 0.0231 | N/A | N/A |
| *b* path X *Y_2_* lag | 0.0073 | N/A | N/A |
| Baseline correlation X *M_2_* lag | 0.0097 | N/A | N/A |
| Baseline correlation X *Y_2_* lag | 0.0152 | N/A | N/A |
|  |  |  |  |
| **Three-way interactions** |  |  |  |
| Stability X *b* path X *Y_2_* lag | 0.0069 | N/A | N/A |
| Baseline correlation X Stability X *M_2_* lag | 0.0069 | N/A | N/A |
| Baseline correlation X Stability X *Y_2_* lag | 0.0064 | N/A | N/A |

Table S7 displays the significant predictors of the Akaike’s Information Criterion (AIC) for the difference score, residualized change score, and cross-sectional models that had semi-partial eta-squared values of 0.005 (within rounding) or greater.

| Table S7 | | | |
| --- | --- | --- | --- |
| *Semi-partial η^2^ for Predictors of AIC* | | | |
| Effect Type/Variables | Difference Score | Residualized Change Score | Cross-sectional |
| **Main Effects** |  |  |  |
| Stability | 0.0817 | 0.0205 | 0.2265 |
| *b* path | 0.0117 | 0.0269 | N/A |
| *M_2_* lag | N/A | 0.0601 | 0.0119 |
| *Y_2_* lag | 0.0327 | 0.2002 | 0.0401 |
| Nobs | 0.6853 | 0.4113 | 0.4804 |
|  |  |  |  |
| **Two-way interactions** |  |  |  |
| Stability X *M_2_* lag | 0.0084 | N/A | N/A |
| Stability X *Y_2_* lag | 0.0161 | N/A | 0.0067 |
| Stability X Nobs | 0.0565 | 0.0138 | 0.1544 |
| *b* path X Stability | 0.0053 | 0.0165 | N/A |
| Nobs X *M_2_* lag | N/A | 0.0415 | 0.0081 |
| Nobs X *Y_2_* lag | 0.0219 | 0.1341 | 0.0273 |
| Nobs X *b* path | 0.0082 | 0.0182 | N/A |
|  |  |  |  |
| **Three-way interactions** |  |  |  |
| Stability X Nobs X *M_2_* lag | 0.0059 | N/A | N/A |
| Stability X Nobs X *Y_2_* lag | 0.0106 | N/A | 0.0045 |
| Stability X Nobs X *b* path | N/A | 0.0120 | N/A |

Table S8 displays the significant predictors of the Bayesian Information Criterion (BIC) for the difference score, residualized change score, and cross-sectional models that had semi-partial eta-squared values of 0.005 (within rounding) or greater.

| Table S8 | | | |
| --- | --- | --- | --- |
| *Semi-partial η^2^ for Predictors of BIC* | | | |
| Effect Type/Variables | Difference Score | Residualized Change Score | Cross-sectional |
| **Main Effects** |  |  |  |
| Stability | 0.0742 | 0.0181 | 0.2088 |
| *b* path | 0.0106 | 0.0238 | N/A |
| *M_2_* lag | N/A | 0.0532 | 0.0110 |
| *Y_2_* lag | 0.0297 | 0.1772 | 0.0370 |
| Nobs | 0.7139 | 0.4782 | 0.5208 |
|  |  |  |  |
| **Two-way interactions** |  |  |  |
| Stability X *M_2_* lag | 0.0076 | N/A | N/A |
| Stability X *Y_2_* lag | 0.0146 | N/A | 0.0061 |
| Stability X Nobs | 0.0513 | 0.0122 | 0.1423 |
| *b* path X Stability | 0.0048 | 0.0146 | N/A |
| Nobs X *M_2_* lag | N/A | 0.0367 | 0.0075 |
| Nobs X *Y_2_* lag | 0.0199 | 0.1187 | 0.0252 |
| Nobs X *b* path | 0.0074 | 0.0161 | N/A |
|  |  |  |  |
| **Three-way interactions** |  |  |  |
| Stability X Nobs X *M_2_* lag | 0.0053 | N/A | N/A |
| Stability X Nobs X *Y_2_* lag | 0.0096 | N/A | N/A |
| Stability X Nobs X *b* path | N/A | 0.0106 | N/A |

**Results for SRMR, AIC, and BIC**

The SRMR is an index of badness of fit (Bentler, 1995; West, Taylor, & Wu, 2012). Lower values indicate better fit than do higher values. The cut-off for good fit is SRMR values less than .08 (Hu & Bentler, 1999; West et al., 2012). The AIC is a model selection index which is used to compare models that are either nested or non-nested (Akaike, 1973; West et al., 2012). When comparing two models, the model with the lower AIC value is selected as the better fitting model. The BIC, like the AIC, is a model selection index (Raferty, 1995; West et al., 2012). When comparing the fit of two models, the model with the lower BIC value is selected as the better fitting model.

**SRMR**

**Difference score model.** There were three-way interactions of stability by the *b_y2m2_* path by *Y_2_* cross-lag (*η^2^* = 0.0069), of baseline correlation by stability by *M_2_* cross-lag (*η^2^* = 0.0069), and of baseline correlation by stability by *Y_2_* cross-lag (*η^2^* = 0.0064). Table S9 displays the SRMR results tabled as a function of the *b_y2m2_* path, baseline correlation, stability, *M_2_* cross-lag and *Y_2_* cross-lag for all models. As stability increased to 1.00 and when both cross-lags = 0.00, the SRMR decreased in magnitude approaching the conventional cut-off value of .08. The SRMR was below the conventional cut-off of .08 when both cross-lags = 0.00, stability = 1.00 and the *b_y2m2_* path was either = 0.00 or 0.14. SRMR values were slightly higher when the baseline correlation was = 0.00 compared to when the baseline correlation was = 0.50 (see Table S9).

**Residualized change score model.** There were no predictors that had semi-partial eta-squared values of 0.01 or greater for the SRMR values for the residualized change score model. When either the *M_2_* cross-lag or the *Y_2_* cross-lag or both were equal to 0.50, the magnitude of the SRMR for the residualized change score model was above the conventional cut-off of .08. When both cross-lags = 0.00 the magnitude of the SRMR was below the conventional cut-off value of 0.95 except for when Stability was = 1.00 and the *b_y2m2_* path was greater than or equal to 0.39 (see Table S9).

**Cross-sectional model.** There were no predictors that had semi-partial eta-squared values of 0.01 or greater for the SRMR values for the cross-sectional model. When either the *M_2_* cross-lag or the *Y_2_* cross-lag or both were equal to 0.50, the magnitude of the SRMR for the cross-sectional model was above the conventional cut-off of .08. When Stability was greater than 0.00 the magnitude of the SRMR values for the cross-sectional model was above the conventional cut-off of .08. When both cross-lags = 0.00 and Stability = 0.00 the values of the SRMR were below the conventional cut-off value of .08 (see Table S9).

| Table S9 | | | | | | | | | | | | | | |
| --- | --- | --- | --- | --- | --- | --- | --- | --- | --- | --- | --- | --- | --- | --- |
| SRMR values for the difference score model (Diff), residualized change score model (Res), and cross-sectional model (Cross). | | | | | | | | | | | | | | |
|  | | | *M_2_* cross-lag | | | | | | | | | | | |
|  |  |  | 0 | | | | | | 0.5 | | | | | |
|  |  |  | *Y_2_* cross-lag | | | | | | *Y_2_* cross-lag | | | | | |
|  |  |  | 0 | | | 0.5 | | | 0 | | | 0.5 | | |
|  |  |  | Diff | Res | Cross | Diff | Res | Cross | Diff | Res | Cross | Diff | Res | Cross |
| Stability | Base. Corr. | *b_y2m2_* path | 0.657 | **0.026** | **0.036** | 0.576 | 0.143 | 0.146 | 0.615 | 0.106 | 0.110 | 0.537 | 0.176 | 0.179 |
| 0 | 0 | 0 |  |  |  |  |  |  |  |  |  |  |  |  |
|  |  | 0.14 | 0.646 | **0.026** | **0.035** | 0.572 | 0.142 | 0.145 | 0.572 | 0.107 | 0.111 | 0.519 | 0.176 | 0.178 |
|  |  | 0.39 | 0.600 | **0.026** | **0.035** | 0.554 | 0.136 | 0.137 | 0.481 | 0.109 | 0.118 | 0.478 | 0.171 | 0.174 |
|  |  | 0.59 | 0.544 | **0.026** | **0.034** | 0.530 | 0.127 | 0.125 | 0.415 | 0.111 | 0.127 | 0.440 | 0.163 | 0.169 |
|  | 0.5 | 0 | 0.813 | **0.023** | **0.035** | 0.526 | 0.109 | 0.162 | 0.670 | 0.089 | 0.133 | 0.339 | 0.148 | 0.206 |
|  |  | 0.14 | 0.797 | **0.023** | **0.035** | 0.524 | 0.108 | 0.160 | 0.601 | 0.090 | 0.135 | 0.327 | 0.146 | 0.205 |
|  |  | 0.39 | 0.725 | **0.023** | **0.035** | 0.512 | 2.815 | 0.604 | 0.441 | 0.092 | 0.145 | 0.322 | 0.146 | 0.206 |
|  |  | 0.59 | 0.637 | **0.023** | **0.034** | 0.498 | 0.096 | 0.139 | 0.314 | 0.094 | 0.157 | 0.320 | 0.145 | 0.206 |
| 0.3 | 0 | 0 | 0.439 | **0.026** | 0.091 | 0.406 | 0.146 | 0.162 | 0.427 | 0.106 | 0.132 | 0.406 | 0.181 | 0.188 |
|  |  | 0.14 | 0.431 | **0.028** | 0.091 | 0.403 | 0.149 | 0.164 | 0.387 | 0.109 | 0.139 | 0.387 | 0.183 | 0.191 |
|  |  | 0.39 | 0.400 | **0.036** | 0.090 | 0.389 | 0.149 | 0.164 | 0.322 | 0.115 | 0.150 | 0.356 | 0.183 | 0.194 |
|  |  | 0.59 | 0.363 | **0.046** | 0.090 | 0.374 | 0.145 | 0.159 | 0.286 | 0.121 | 0.159 | 0.333 | 0.180 | 0.194 |
|  | 0.5 | 0 | 0.539 | **0.023** | 0.105 | 0.327 | 0.103 | 0.204 | 0.437 | 0.086 | 0.184 | 0.204 | 0.152 | 0.253 |
|  |  | 0.14 | 0.512 | **0.025** | 0.109 | 0.324 | 0.105 | 0.205 | 0.350 | 0.089 | 0.195 | 0.189 | 0.153 | 0.260 |
|  |  | 0.39 | 0.445 | **0.030** | 0.115 | 0.319 | 0.107 | 0.203 | 0.212 | 0.096 | 0.213 | 0.181 | 0.152 | 0.267 |
|  |  | 0.59 | 0.381 | **0.037** | 0.117 | 0.314 | 0.105 | 0.196 | 0.144 | 0.102 | 0.225 | 0.187 | 0.149 | 0.269 |
| 1 | 0 | 0 | **0.038** | **0.024** | 0.217 | 0.145 | 0.139 | 0.232 | 0.106 | 0.099 | 0.224 | 0.190 | 0.184 | 0.238 |
|  |  | 0.14 | **0.047** | **0.035** | 0.216 | 0.159 | 0.153 | 0.234 | 0.115 | 0.110 | 0.228 | 0.196 | 0.193 | 0.243 |
|  |  | 0.39 | 0.082 | **0.074** | 0.215 | 0.178 | 0.173 | 0.236 | 0.138 | 0.133 | 0.233 | 0.206 | 0.204 | 0.247 |
|  |  | 0.59 | 0.115 | 0.109 | 0.214 | 0.190 | 0.185 | 0.236 | 0.159 | 0.153 | 0.235 | 0.211 | 0.210 | 0.248 |
|  | 0.5 | 0 | **0.037** | **0.021** | 0.250 | 0.143 | 0.091 | 0.288 | 0.122 | 0.078 | 0.284 | 0.185 | 0.135 | 0.315 |
|  |  | 0.14 | **0.046** | **0.028** | 0.257 | 0.154 | 0.099 | 0.291 | 0.132 | 0.087 | 0.294 | 0.197 | 0.142 | 0.320 |
|  |  | 0.39 | 0.082 | **0.051** | 0.266 | 0.170 | 0.111 | 0.292 | 0.164 | 0.103 | 0.306 | 0.214 | 0.148 | 0.324 |
|  |  | 0.59 | 0.113 | **0.072** | 0.268 | 0.181 | 0.119 | 0.291 | 0.189 | 0.116 | 0.311 | 0.225 | 0.152 | 0.325 |
| *Note.* SRMR values less than or equal to .08 are bolded and underlined. | | | | | | | | | | | | | | |

**AIC**

The AIC is used as a model selection index. When two models are compared to each other, the model with the lower AIC is selected as the better fitting model. The AIC values for the difference score model, the residualized change score model, and the cross-sectional model were each compared to the AIC values of the ANCOVA model. The average AIC value for the ANCOVA model equaled 40 therefore anytime either the difference score, residualized change score, or cross-sectional models resulted in average AIC values lower than 40, they were deemed better fitting models compared to the ANCOVA model (bolded and underlined entries in Table S10).

**Difference score model.** There were three-way interactions of stability by sample size (*Nobs*) by *M_2_* cross-lag (*η^2^* = 0.0059), and of stability by sample size (*Nobs*) by *Y_2_* cross-lag (*η^2^* = 0.0106). Table S10 displays the AIC results tabled as a function of the *b_y2m2_* path, stability, sample size (*N*), *M_2_* cross-lag and *Y_2_* cross-lag for all models. As stability increased to 1.00 and when both cross-lags = 0.00, the AIC values for the difference score model fell below the AIC values for the ANCOVA model for sample sizes of 50 and 100 when the *b_y2m2_* path was less than or equal to 0.14 and for sample sizes of 200 and larger when the *b_y2m2_* path = 0.00 (see Table S10).

**Residualized change score model.** There were three-way interactions of stability by sample size by the *b_y2m2_* path (*η^2^* = 0.0106). When either the *M_2_* cross-lag or the *Y_2_* cross-lag or both were equal to 0.50, the AIC values for the residualized change score model were higher than the AIC values for the ANCOVA model. When both cross-lags = 0.00 and stability = 0.00, the AIC values for the residualized change score model were lower than the AIC values for the ANCOVA model. As sample size, stability, and the *b_y2m2_* path increased in magnitude, the AIC values of the residualized change score model became higher than the AIC values for the ANCOVA model (see Table S8).

**Cross-sectional model.** There was a three-way interaction of stability by sample size by *b_y2m2_* path (*η^2^* = 0.0045). When either the *M_2_* cross-lag or the *Y_2_* cross-lag or both were equal to 0.50, the AIC values for the cross-sectional model were higher than the AIC values for the ANCOVA model. When stability was greater than 0.00 the AIC values for the cross-sectional model were higher than the AIC values for the ANCOVA model. When both cross-lags = 0.00 and stability = 0.00 the AIC values for the cross-sectional model were lower than the AIC values for the ANCOVA model (see Table S10).

| Table S10 | | | | | | | | | | | | | | |
| --- | --- | --- | --- | --- | --- | --- | --- | --- | --- | --- | --- | --- | --- | --- |
| AIC values for the difference score model (Diff), residualized change score model (Res), and cross-sectional model (Cross). | | | | | | | | | | | | | | |
|  | | | *M_2_* cross-lag | | | | | | | | | | | |
|  |  |  | 0 | | | | | | 0.5 | | | | | |
|  |  |  | *Y_2_* cross-lag | | | | | | *Y_2_* cross-lag | | | | | |
|  |  |  | 0 | | | 0.5 | | | 0 | | | 0.5 | | |
|  |  |  | Diff | Res | Cross | Diff | Res | Cross | Diff | Res | Cross | Diff | Res | Cross |
| Stability | Nobs | *b_y2m2_* path | 106.2 | **34.5** | **36.3** | 109.1 | 59.8 | 64.3 | 106.8 | 48.6 | 52.2 | 100.1 | 73.1 | 79.4 |
| 0 | 50 | 0 |  |  |  |  |  |  |  |  |  |  |  |  |
|  |  | 0.14 | 105.6 | **34.6** | **36.4** | 111.3 | 60.7 | 64.2 | 104.1 | 49.3 | 52.2 | 101.3 | 74.6 | 79.6 |
|  |  | 0.39 | 105.3 | **34.9** | **36.3** | 115.8 | 62.3 | 64.2 | 100.5 | 51.5 | 52.4 | 104.2 | 75.9 | 79.4 |
|  |  | 0.59 | 107.3 | **35.7** | **36.3** | 122.3 | 64.4 | 64.2 | 99.4 | 55.6 | 52.2 | 108.4 | 78.4 | 79.4 |
|  | 100 | 0 | 177.3 | **34.4** | **36.2** | 183.4 | 85.6 | 92.5 | 178.5 | 62.6 | 68.2 | 165.0 | 112.6 | 123.0 |
|  |  | 0.14 | 176.2 | **34.5** | **36.1** | 187.0 | 86.4 | 92.3 | 173.2 | 64.1 | 68.3 | 167.3 | 113.7 | 123.2 |
|  |  | 0.39 | 175.8 | **34.8** | **36.1** | 196.5 | 88.4 | 92.2 | 165.9 | 67.5 | 68.3 | 173.3 | 116.9 | 123.3 |
|  |  | 0.59 | 180.0 | **35.5** | **36.1** | 209.7 | 91.4 | 92.4 | 163.8 | 76.2 | 68.5 | 181.6 | 120.8 | 123.2 |
|  | 200 | 0 | 319.6 | **34.4** | **36.1** | 331.9 | 136.8 | 149.3 | 322.4 | 91.2 | 100.9 | 294.9 | 190.4 | 210.6 |
|  |  | 0.14 | 317.1 | **34.4** | **36.1** | 339.9 | 138.2 | 149.2 | 311.5 | 92.9 | 100.8 | 299.6 | 192.9 | 211.0 |
|  |  | 0.39 | 317.1 | **34.7** | **36.1** | 359.0 | 141.0 | 148.9 | 297.0 | 100.6 | 101.0 | 311.6 | 197.6 | 210.9 |
|  |  | 0.59 | 325.1 | **35.5** | **36.1** | 385.3 | 145.2 | 149.1 | 292.9 | 117.0 | 100.8 | 328.5 | 205.6 | 211.1 |
|  | 500 | 0 | 747.8 | **34.5** | **36.1** | 776.9 | 290.6 | 319.8 | 754.1 | 176.9 | 198.2 | 685.8 | 426.1 | 474.0 |
|  |  | 0.14 | 740.6 | **34.4** | **36.0** | 797.3 | 292.7 | 319.6 | 726.4 | 180.3 | 198.4 | 697.4 | 430.8 | 474.3 |
|  |  | 0.39 | 741.2 | **34.7** | **36.1** | 846.7 | 299.9 | 319.9 | 690.6 | 199.8 | 198.5 | 727.4 | 440.9 | 474.1 |
|  |  | 0.59 | 761.6 | **35.4** | **36.0** | 911.8 | 303.8 | 319.9 | 679.2 | 241.1 | 198.7 | 770.2 | 458.6 | 474.1 |
| 0.3 | 50 | 0 | 77.8 | **34.5** | 45.0 | 85.2 | 59.8 | 72.4 | 80.9 | 48.7 | 61.7 | 83.3 | 73.5 | 85.7 |
|  |  | 0.14 | 76.9 | **34.6** | 45.0 | 88.9 | 62.0 | 72.4 | 78.5 | 49.1 | 61.6 | 87.0 | 76.3 | 85.7 |
|  |  | 0.39 | 77.8 | **35.7** | 45.1 | 97.0 | 66.2 | 72.4 | 77.1 | 52.3 | 61.6 | 96.2 | 82.8 | 85.6 |
|  |  | 0.59 | 82.3 | **37.9** | 45.0 | 106.7 | 70.9 | 72.4 | 80.2 | 58.7 | 61.6 | 107.7 | 90.7 | 85.7 |
|  | 100 | 0 | 119.9 | **34.4** | 53.7 | 135.2 | 85.4 | 109.4 | 126.5 | 62.9 | 87.2 | 131.1 | 112.7 | 135.9 |
|  |  | 0.14 | 118.3 | **34.6** | 53.7 | 142.3 | 89.6 | 109.4 | 121.6 | 63.7 | 87.2 | 138.8 | 118.4 | 135.7 |
|  |  | 0.39 | 119.9 | **36.3** | 53.7 | 158.7 | 97.8 | 109.5 | 118.3 | 69.7 | 87.1 | 157.2 | 131.3 | 135.9 |
|  |  | 0.59 | 129.1 | 40.1 | 53.7 | 178.6 | 107.1 | 109.4 | 125.0 | 82.4 | 87.2 | 180.3 | 146.9 | 135.9 |
|  | 200 | 0 | 204.2 | **34.4** | 71.5 | 234.7 | 136.4 | 183.2 | 216.8 | 91.1 | 138.7 | 226.6 | 191.1 | 236.4 |
|  |  | 0.14 | 200.4 | **34.8** | 71.5 | 249.5 | 144.7 | 183.1 | 207.7 | 93.1 | 138.7 | 242.4 | 202.9 | 236.4 |
|  |  | 0.39 | 204.6 | **37.9** | 71.3 | 282.3 | 161.2 | 183.1 | 201.2 | 105.2 | 138.7 | 279.4 | 228.7 | 236.5 |
|  |  | 0.59 | 222.5 | 44.4 | 71.4 | 322.1 | 179.6 | 183.3 | 214.4 | 129.9 | 138.6 | 325.2 | 259.8 | 236.3 |
|  | 500 | 0 | 457.7 | **34.3** | 124.9 | 533.9 | 289.6 | 404.9 | 490.1 | 177.2 | 294.0 | 513.4 | 426.8 | 538.3 |
|  |  | 0.14 | 449.6 | **35.3** | 124.8 | 571.4 | 310.7 | 404.9 | 465.9 | 181.1 | 293.6 | 553.4 | 456.6 | 538.5 |
|  |  | 0.39 | 457.7 | 42.5 | 125.1 | 653.7 | 352.2 | 405.4 | 449.9 | 210.9 | 293.2 | 645.8 | 520.4 | 538.1 |
|  |  | 0.59 | 503.6 | 58.2 | 124.8 | 753.0 | 397.1 | 404.5 | 483.2 | 273.1 | 293.6 | 761.1 | 598.5 | 537.9 |
| 1 | 50 | 0 | 36.3 | **34.5** | 106.2 | 64.1 | 59.8 | 118.8 | 52.3 | 48.7 | 113.6 | 79.1 | 73.2 | 117.9 |
|  |  | 0.14 | 37.3 | **35.5** | 106.2 | 71.3 | 66.5 | 119.0 | 53.3 | 50.1 | 113.6 | 86.2 | 80.6 | 118.0 |
|  |  | 0.39 | 44.4 | 42.1 | 106.4 | 84.8 | 79.3 | 119.0 | 60.0 | 59.0 | 113.7 | 99.3 | 95.2 | 117.9 |
|  |  | 0.59 | 57.5 | 54.2 | 106.2 | 99.0 | 93.0 | 118.9 | 72.8 | 74.8 | 113.7 | 113.6 | 111.1 | 117.9 |
|  | 100 | 0 | 36.2 | **34.4** | 177.5 | 105.4 | 96.8 | 202.9 | 68.3 | 62.8 | 192.6 | 129.9 | 119.2 | 199.9 |
|  |  | 0.14 | **38.2** | **36.3** | 177.3 | 117.3 | 108.0 | 203.0 | 70.4 | 65.5 | 192.6 | 138.6 | 128.9 | 200.8 |
|  |  | 0.39 | 52.6 | 49.3 | 177.6 | 139.5 | 129.3 | 202.9 | 84.2 | 83.8 | 192.3 | 163.8 | 156.3 | 201.0 |
|  |  | 0.59 | 78.9 | 73.4 | 177.2 | 162.7 | 151.8 | 202.9 | 109.8 | 115.4 | 192.2 | 189.8 | 185.8 | 200.4 |
|  | 200 | 0 | **36.0** | **34.3** | 319.6 | 149.1 | 136.3 | 371.0 | 100.9 | 91.5 | 350.2 | 210.6 | 191.0 | 367.6 |
|  |  | 0.14 | 40.2 | **38.0** | 320.1 | 178.1 | 163.1 | 371.3 | 104.8 | 96.7 | 350.1 | 239.0 | 221.2 | 367.8 |
|  |  | 0.39 | 69.1 | 63.9 | 319.9 | 233.0 | 214.8 | 371.5 | 132.8 | 133.3 | 349.9 | 293.0 | 280.6 | 367.6 |
|  |  | 0.59 | 122.2 | 112.0 | 320.0 | 290.0 | 269.2 | 370.9 | 183.6 | 196.1 | 350.1 | 349.5 | 344.1 | 367.8 |
|  | 500 | 0 | **36.0** | **34.3** | 747.5 | 319.9 | 290.0 | 877.1 | 198.2 | 177.0 | 823.5 | 474.0 | 427.0 | 868.0 |
|  |  | 0.14 | 46.4 | 43.4 | 747.2 | 392.2 | 356.9 | 876.7 | 208.5 | 190.6 | 824.0 | 544.4 | 501.6 | 867.4 |
|  |  | 0.39 | 119.0 | 108.0 | 747.9 | 529.7 | 486.1 | 876.9 | 278.4 | 281.5 | 823.4 | 680.2 | 651.6 | 868.1 |
|  |  | 0.59 | 251.6 | 228.0 | 747.7 | 673.8 | 623.2 | 876.6 | 406.6 | 440.5 | 824.2 | 822.1 | 810.5 | 867.4 |
| *Note.* The AIC for the ANCOVA model was equal to 40. Bolded and underlined AIC values indicate when the respective model had a lower AIC value than the ANCOVA model on average. | | | | | | | | | | | | | | |

**BIC**

The BIC is used as a model selection index. When two models are compared to each other, the model with the lower AIC is selected as the better fitting model. The BIC values for the difference score model, the residualized change score model, and the cross-sectional model were each compared to the BIC values of the ANCOVA model. Because BIC varies with sample size, the BIC for the difference score, residualized change score, and cross-sectional models were compared to the BIC for the ANCOVA model for each sample size. The average BIC value for the ANCOVA model for *N* = 50 was 78.24, for *N* = 100 was 92.10, for *N* = 200 was 105.97, and for *N* = 500 was 124.29. Anytime the difference score, residualized change score, or cross-sectional models resulted in average BIC values lower than the ANCOVA BIC for the respective sample sizes, they were deemed better fitting models compared to the ANCOVA model (entries bolded and underlined in Table S11).

**Difference score model.** There were three-way interactions of stability by sample size by *M_2_* cross-lag (*η^2^* = 0.0053) and of stability by sample Size by *Y_2_* cross-lag (*η^2^* = 0.0096). Table S11 displays the BIC results tabled as a function of the *b_y2m2_* path, sample size, stability, *M_2_* cross-lag and *Y_2_* cross-lag for all models. As stability increased to 1.00 and when both cross-lags = 0.00, the BIC values for the difference score model fell below the BIC values for the ANCOVA model. The BIC values for the difference score model fell below the BIC values for the ANCOVA model for sample sizes of 50 and 100 when the *b_y2m2_* path was less than or equal to 0.39 and for sample sizes of 500 and larger when the *b_y2m2_* path was less than or equal to 0.14 (see Table S11).

**Residualized change score model.** There were three-way interactions of stability by sample size by the *b_y2m2_ path* (*η^2^* = 0.0106). When either the *M_2_* cross-lag or the *Y_2_* cross-lag or both were equal to 0.50, the BIC values for the residualized change score model were higher than the BIC values for the ANCOVA model. When both cross-lags = 0.00 and Stability = 0.00, the BIC values for the residualized change score model were lower than the BIC values for the ANCOVA model. As sample size, stability, and the *b_y2m2_* path increased in magnitude, the BIC values of the residualized change score model became higher than the BIC values for the ANCOVA model (see Table S11).

**Cross-sectional model.** There were two-way interactions of stability by sample size (*η^2^* = 0.1423), of stability by *Y_2_* cross-lag (*η^2^* = 0.0061), of sample size by *M_2_* cross-lag (*η^2^* = 0.0075), and of sample size by *Y_2_* cross-lag (*η^2^* = 0.0252). When either the *M_2_* cross-lag or the *Y_2_* cross-lag or both were equal to 0.50, the BIC values for the cross-sectional model were higher than the BIC values for the ANCOVA model. When both cross-lags = 0.00 and stability = 0.00 the BIC values for the cross-sectional model were lower than the BIC values for the ANCOVA model. When stability was greater than 0.30 for samples size of 50 and 100, the BIC values for the cross-sectional model were lower than the BIC values for the ANCOVA model (see Table S11).

| Table S11 | | | | | | | | | | | | | | |
| --- | --- | --- | --- | --- | --- | --- | --- | --- | --- | --- | --- | --- | --- | --- |
| BIC values for the difference score model (Diff), residualized change score model (Res), and cross-sectional model (Cross). | | | | | | | | | | | | | | |
|  | | | *M_2_* cross-lag | | | | | | | | | | | |
|  |  |  | 0 | | | | | | 0.5 | | | | | |
|  |  |  | *Y_2_* cross-lag | | | | | | *Y_2_* cross-lag | | | | | |
|  |  |  | 0 | | | 0.5 | | | 0 | | | 0.5 | | |
|  |  |  | Diff | Res | Cross | Diff | Res | Cross | Diff | Res | Cross | Diff | Res | Cross |
| Stability | Nobs | *b_y2m2_* path | 136.8 | **65.1** | **66.9** | 139.7 | **90.4** | **94.9** | 137.4 | **79.2** | **82.8** | 130.7 | 103.7 | 110.0 |
| 0 | 50 | 0 |  |  |  |  |  |  |  |  |  |  |  |  |
|  |  | 0.14 | 136.2 | **65.2** | **66.9** | 141.9 | **91.3** | **94.8** | 134.7 | **79.9** | **82.8** | 131.9 | 105.2 | 110.2 |
|  |  | 0.39 | 135.9 | **65.5** | **66.9** | 146.4 | **92.9** | **94.8** | 131.1 | **82.1** | **82.9** | 134.8 | 106.5 | 109.9 |
|  |  | 0.59 | 137.9 | **66.3** | **66.9** | 152.9 | **95.0** | **94.8** | 130.0 | **86.2** | **82.8** | 138.9 | 109.0 | 110.0 |
|  | 100 | 0 | 218.9 | **76.1** | **77.8** | 225.0 | 127.2 | 134.2 | 220.2 | 104.3 | 109.9 | 206.7 | 154.3 | 164.7 |
|  |  | 0.14 | 217.9 | **76.1** | **77.8** | 228.7 | 128.1 | 133.9 | 214.9 | 105.7 | 110.0 | 209.0 | 155.4 | 164.9 |
|  |  | 0.39 | 217.4 | **76.5** | **77.8** | 238.2 | 130.1 | 133.8 | 207.6 | 109.2 | 110.0 | 215.0 | 158.6 | 165.0 |
|  |  | 0.59 | 221.7 | **77.2** | **77.8** | 251.4 | 133.1 | 134.1 | 205.5 | 117.9 | 110.2 | 223.2 | 162.5 | 164.9 |
|  | 200 | 0 | 372.4 | **87.2** | **88.9** | 384.7 | 189.6 | 202.1 | 375.2 | 143.9 | 153.7 | 347.7 | 243.2 | 263.4 |
|  |  | 0.14 | 369.9 | **87.2** | **88.8** | 392.6 | 191.0 | 202.0 | 364.3 | 145.6 | 153.6 | 352.4 | 245.6 | 263.8 |
|  |  | 0.39 | 369.9 | **87.5** | **88.9** | 411.7 | 193.8 | 201.7 | 349.8 | 153.4 | 153.8 | 364.4 | 250.4 | 263.7 |
|  |  | 0.59 | 377.9 | **88.2** | **88.8** | 438.1 | 197.9 | 201.9 | 345.6 | 169.8 | 153.6 | 381.2 | 258.4 | 263.8 |
|  | 500 | 0 | 815.2 | **101.9** | **103.5** | 844.3 | 358.1 | 387.2 | 821.5 | 244.3 | 265.7 | 753.2 | 493.5 | 541.4 |
|  |  | 0.14 | 808.0 | **101.8** | **103.4** | 864.7 | 360.1 | 387.0 | 793.9 | 247.7 | 265.8 | 764.8 | 498.2 | 541.8 |
|  |  | 0.39 | 808.6 | **102.2** | **103.5** | 914.2 | 367.4 | 387.3 | 758.0 | 267.2 | 265.9 | 794.8 | 508.3 | 541.5 |
|  |  | 0.59 | 829.0 | **102.9** | **103.4** | 979.2 | 371.2 | 387.3 | 746.6 | 308.5 | 266.1 | 837.6 | 526.1 | 541.6 |
| 0.3 | 50 | 0 | 108.4 | **65.1** | **75.6** | 115.8 | **90.4** | 103.0 | 111.5 | **79.3** | **92.3** | 113.9 | 104.1 | 116.3 |
|  |  | 0.14 | 107.5 | **65.2** | **75.6** | 119.5 | **92.6** | 103.0 | 109.1 | **79.7** | **92.2** | 117.6 | 106.9 | 116.3 |
|  |  | 0.39 | 108.4 | **66.3** | **75.7** | 127.5 | **96.8** | 103.0 | 107.7 | **82.9** | **92.2** | 126.8 | 113.4 | 116.2 |
|  |  | 0.59 | 112.9 | **68.5** | **75.6** | 137.2 | 101.5 | 103.0 | 110.8 | **89.3** | **92.2** | 138.3 | 121.3 | 116.3 |
|  | 100 | 0 | 161.6 | **76.1** | **95.4** | 176.9 | 127.1 | 151.1 | 168.2 | 104.6 | 128.9 | 172.7 | 154.4 | 177.5 |
|  |  | 0.14 | 160.0 | **76.3** | **95.4** | 183.9 | 131.2 | 151.0 | 163.3 | 105.4 | 128.9 | 180.4 | 160.1 | 177.3 |
|  |  | 0.39 | 161.6 | **78.0** | **95.4** | 200.4 | 139.5 | 151.2 | 160.0 | 111.4 | 128.8 | 198.9 | 173.0 | 177.6 |
|  |  | 0.59 | 170.8 | **81.8** | **95.4** | 220.3 | 148.7 | 151.1 | 166.7 | 124.1 | 128.8 | 222.0 | 188.6 | 177.6 |
|  | 200 | 0 | 256.9 | **87.1** | 124.2 | 287.5 | 189.1 | 236.0 | 269.6 | 143.9 | 191.5 | 279.3 | 243.9 | 289.2 |
|  |  | 0.14 | 253.2 | **87.5** | 124.3 | 302.2 | 197.5 | 235.9 | 260.5 | 145.8 | 191.5 | 295.2 | 255.7 | 289.1 |
|  |  | 0.39 | 257.4 | **90.6** | 124.1 | 335.1 | 214.0 | 235.9 | 254.0 | 157.9 | 191.5 | 332.2 | 281.5 | 289.3 |
|  |  | 0.59 | 275.2 | **97.2** | 124.1 | 374.9 | 232.3 | 236.1 | 267.2 | 182.7 | 191.4 | 378.0 | 312.5 | 289.1 |
|  | 500 | 0 | 525.2 | **101.7** | 192.3 | 601.4 | 357.0 | 472.3 | 557.6 | 244.7 | 361.4 | 580.9 | 494.2 | 605.7 |
|  |  | 0.14 | 517.0 | **102.7** | 192.2 | 638.9 | 378.2 | 472.3 | 533.4 | 248.5 | 361.0 | 620.8 | 524.1 | 605.9 |
|  |  | 0.39 | 525.2 | **110.0** | 192.5 | 721.1 | 419.6 | 472.8 | 517.4 | 278.4 | 360.7 | 713.2 | 587.8 | 605.6 |
|  |  | 0.59 | 571.1 | 125.6 | 192.2 | 820.4 | 464.5 | 471.9 | 550.6 | 340.5 | 361.0 | 828.6 | 666.0 | 605.4 |
| 1 | 50 | 0 | **66.9** | **65.1** | 136.8 | 94.7 | **90.4** | 149.4 | **82.8** | **79.3** | 144.2 | 109.7 | 103.8 | 148.5 |
|  |  | 0.14 | **67.9** | **66.1** | 136.8 | 101.9 | **97.1** | 149.6 | **83.9** | **80.7** | 144.2 | 116.8 | 111.2 | 148.6 |
|  |  | 0.39 | **75.0** | **72.7** | 137.0 | 115.4 | 109.9 | 149.6 | **90.6** | **89.6** | 144.3 | 129.9 | 125.8 | 148.5 |
|  |  | 0.59 | **88.0** | **84.8** | 136.8 | 129.6 | 123.6 | 149.5 | 103.4 | 105.4 | 144.3 | 144.2 | 141.7 | 148.5 |
|  | 100 | 0 | **77.8** | **76.1** | 219.2 | 147.1 | 138.5 | 244.5 | 110.0 | 104.5 | 234.3 | 171.6 | 160.9 | 241.6 |
|  |  | 0.14 | **79.9** | **77.9** | 219.0 | 158.9 | 149.7 | 244.6 | 112.0 | 107.2 | 234.3 | 180.3 | 170.6 | 242.4 |
|  |  | 0.39 | **94.3** | **91.0** | 219.2 | 181.2 | 171.0 | 244.6 | 125.9 | 125.5 | 234.0 | 205.4 | 198.0 | 242.7 |
|  |  | 0.59 | 120.6 | 115.1 | 218.9 | 204.4 | 193.5 | 244.6 | 151.5 | 157.0 | 233.9 | 231.5 | 227.5 | 242.1 |
|  | 200 | 0 | **88.8** | **87.1** | 372.3 | 201.9 | 189.1 | 423.7 | 153.7 | 144.3 | 403.0 | 263.4 | 243.8 | 420.3 |
|  |  | 0.14 | **93.0** | **90.8** | 372.9 | 230.9 | 215.9 | 424.1 | 157.6 | 149.5 | 402.8 | 291.8 | 273.9 | 420.6 |
|  |  | 0.39 | 121.9 | 116.7 | 372.6 | 285.8 | 267.6 | 424.3 | 185.6 | 186.0 | 402.7 | 345.7 | 333.3 | 420.4 |
|  |  | 0.59 | 175.0 | 164.8 | 372.8 | 342.8 | 322.0 | 423.7 | 236.4 | 248.9 | 402.9 | 402.3 | 396.9 | 420.6 |
|  | 500 | 0 | **103.5** | **101.7** | 814.9 | 387.3 | 357.4 | 944.5 | 265.6 | 244.4 | 891.0 | 541.4 | 494.4 | 935.4 |
|  |  | 0.14 | **113.8** | **110.8** | 814.6 | 459.6 | 424.3 | 944.1 | 275.9 | 258.1 | 891.4 | 611.8 | 569.1 | 934.9 |
|  |  | 0.39 | 186.4 | 175.4 | 815.3 | 597.2 | 553.5 | 944.3 | 345.8 | 348.9 | 890.8 | 747.6 | 719.0 | 935.5 |
|  |  | 0.59 | 319.0 | 295.4 | 815.2 | 741.3 | 690.6 | 944.0 | 474.0 | 507.9 | 891.6 | 889.5 | 878.0 | 934.8 |
| *Note.* The BIC for the ANCOVA model was equal to 78.24 for *N* = 50, 92.10 for *N* = 100, 105.97 for *N* = 200, and 124.29 for *N* = 500. Bolded and underlined BIC values indicate when the respective model had a lower BIC value than the ANCOVA model on average. | | | | | | | | | | | | | | |

Overall, the model results presented in this supplement match similar patterns to the fit indexes described in the main manuscript.

**R Code**

#install.packages("lavaan")

#install.packages("RMediation")

library(lavaan)

library(RMediation)

#Path to the file exmp.csv must be specified.

#This is a simulated dataset to use to run the code.

#It is NOT the same dataset that appears in the empirical example.

mydata<-read.csv("/exmp.csv",header=TRUE)

N=nrow(mydata)

#Regression approach

#Creating difference scores.

mydata$mdiff<-mydata$m2-mydata$m1

mydata$ydiff<-mydata$y2-mydata$y1

#Creating residualized change scores.

lm1<-lm(m2~m1, data=mydata)

summary(lm1)

im2<-lm1$coefficients[1]

bm2m1<-lm1$coefficients[2]

lm2<-lm(y2~y1, data=mydata)

summary(lm2)

iy2<-lm2$coefficients[1]

by2y1<-lm2$coefficients[2]

mydata$mres<-mydata$m2-(im2+bm2m1*mydata$m1)

mydata$yres<-mydata$y2-(iy2+by2y1*mydata$y1)

#Estimating linear regressions for ANCOVA model.

m2<-lm(m2~x + m1 + y1, data=mydata)

summary(m2)

y2<-lm(y2~x + m2 + y1 + m1, data=mydata)

summary(y2)

#Saving the a path coefficient and standard error;

a<-m2$coefficients[2]

ses<-summary(m2)$coefficients[,2]

sea<-ses[2]

#Saving the b path coefficient and standard error;

b<-y2$coefficients[3]

ses<-summary(y2)$coefficients[,2]

seb<-ses[3]

#Using the saved regression coefficients and standard errors;

distrprodCI<-medci(a,b,sea,seb,rho=0,alpha=0.05,plot=FALSE,plotCI=FALSE,type="dop")

distrprodCI

#Estimating linear regressions with change scores.

diffm<-lm(mdiff~x, data=mydata)

summary(diffm)

diffy<-lm(ydiff~x +mdiff, data=mydata)

summary(diffy)

#Saving the a path coefficient and standard error;

adiff<-diffm$coefficients[2]

ses<-summary(diffm)$coefficients[,2]

seadiff<-ses[2]

#Saving the b path coefficient and standard error;

bdiff<-diffy$coefficients[3]

ses<-summary(diffy)$coefficients[,2]

sebdiff<-ses[3]

#Using the saved regression coefficients and standard errors;

diffdistrprodCI<-medci(adiff,bdiff,seadiff,sebdiff,rho=0,alpha=0.05,plot=FALSE,plotCI=FALSE,type="dop")

diffdistrprodCI

#Estimating linear regressions with residualized change scores.

resm<-lm(mres~x, data=mydata)

summary(resm)

resy<-lm(yres~x +mres, data=mydata)

summary(resy)

#Saving the a path coefficient and standard error;

ares<-resm$coefficients[2]

ses<-summary(resm)$coefficients[,2]

seares<-ses[2]

#Saving the b path coefficient and standard error;

bres<-resy$coefficients[3]

ses<-summary(resy)$coefficients[,2]

sebres<-ses[3]

#Using the saved regression coefficients and standard errors;

resdistrprodCI<-medci(ares,bres,seares,sebres,rho=0,alpha=0.05,plot=FALSE,plotCI=FALSE,type="dop")

resdistrprodCI

#Estimating linear regressions for cross-sectional model.

crossm<-lm(m2~x, data=mydata)

summary(crossm)

crossy<-lm(y2~x +m2, data=mydata)

summary(crossy)

#Saving the a path coefficient and standard error;

across<-crossm$coefficients[2]

ses<-summary(crossm)$coefficients[,2]

seacross<-ses[2]

#Saving the b path coefficient and standard error;

bcross<-crossy$coefficients[3]

ses<-summary(crossy)$coefficients[,2]

sebcross<-ses[3]

#Using the saved regression coefficients and standard errors;

crossdistrprodCI<-medci(across,bcross,seacross,sebcross,rho=0,alpha=0.05,plot=FALSE,plotCI=FALSE,type="dop")

crossdistrprodCI

#LCS specification of two-wave models.

ancova_syntax <-

'

#Defining change in M as a function of M1 and M2

deltam =~ 1*m2

deltam ~~ deltam

deltam ~ 1

m2 ~ 1*m1

m2 ~~ 0*m1

m2 ~~ 0*m2

m2 ~ 0*1

m1 ~ 1

#Defining the change in Y as a function of Y1 and Y2

deltay =~ 1*y2

deltay ~~ deltay

deltay ~ 1

y2 ~ 1*y1

y2 ~~ 0*y1

y2 ~~ 0*y2

y2 ~ 0*1

y1 ~ 1

#Estimating the Pretest correlation between M1 and Y1 and Variance of X

m1 ~~ y1

# Estimated covariance between M1 and X and Y1 and X because these covariances may not be equal to zero especially if X is not a randomized experiment

# without these the model has 2 degrees of freedom (covariances are only constrained to zero) but ANCOVA model should start out as saturated and have 0 degrees of freedom

m1 ~~ x # these covariances may not be equal to zero especially if X is not a randomized experiment

x ~~ y1 # these covariances may not be equal to zero especially if X is not a randomized experiment

#Regression of change in M on X and pretest measures

deltam ~ am2x*x + sm1*m1 + y1

#Regression of change in Y on X, change in M, and pretest measures

deltay ~ x + by2m2*deltam + b*m1 + sy1*y1

#Making constraints to match estimates to ANCOVA

#Estimate of effect of M1 on M2 in ANCOVA

sm := sm1+1

#Estimate of effect of Y1 on Y2 in ANCOVA

sy := sy1+1

#Estimate of effect of M1 on Y2 in ANCOVA

by2m1 := b-by2m2

#Estimate of mediated effect

med := am2x*by2m2

'

ancova <- lavaan::sem(model = ancova_syntax, data = mydata, fixed.x = FALSE)

summary(ancova, standardized=FALSE, fit.measures=TRUE)

lcsa<-ancova@ParTable[["est"]][20]

lcsb<-ancova@ParTable[["est"]][24]

lcssea<-ancova@ParTable[["se"]][20]

lcsseb<-ancova@ParTable[["se"]][24]

#Using the saved regression coefficients and standard errors;

lcsdistrprodCI<-medci(lcsa,lcsb,lcssea,lcsseb,rho=0,alpha=0.05,plot=FALSE,plotCI=FALSE,type="dop")

lcsdistrprodCI

diff_syntax <-

'

#Defining change in M as a function of M1 and M2

deltam =~ 1*m2

deltam ~~ deltam

deltam ~ 1

m2 ~ 1*m1

m2 ~~ 0*m1

m2 ~~ 0*m2

m2 ~ 0*1

m1 ~ 1

#Defining the change in Y as a function of Y1 and Y2

deltay =~ 1*y2

deltay ~~ deltay

deltay ~ 1

y2 ~ 1*y1

y2 ~~ 0*y1

y2 ~~ 0*y2

y2 ~ 0*1

y1 ~ 1

#Estimating the Pretest correlation between M1 and Y1 and Variance of X

m1 ~~ y1

# Estimated covariance between M1 and X and Y1 and X because these covariances may not be equal to zero especially if X is not a randomized experiment

# without these the model has 2 degrees of freedom (covariances are only constrained to zero) but ANCOVA model should start out as saturated and have 0 degrees of freedom

m1 ~~ x # these covariances may not be equal to zero especially if X is not a randomized experiment

x ~~ y1 # these covariances may not be equal to zero especially if X is not a randomized experiment

#Regression of change in M on X and pretest measures

deltam ~ am2x*x

#Regression of change in Y on X, change in M, and pretest measures

deltay ~ x + by2m2*deltam

#Estimate of mediated effect

med := am2x*by2m2

'

diff <- lavaan::sem(model = diff_syntax, data = mydata, fixed.x = FALSE)

summary(diff, standardized=FALSE, fit.measures=TRUE)

lcsadiff<-diff@ParTable[["est"]][20]

lcsbdiff<-diff@ParTable[["est"]][22]

lcsseadiff<-diff@ParTable[["se"]][20]

lcssebdiff<-diff@ParTable[["se"]][22]

#Using the saved regression coefficients and standard errors;

lcsdiffdistrprodCI<-medci(lcsadiff,lcsbdiff,lcsseadiff,lcssebdiff,rho=0,alpha=0.05,plot=FALSE,plotCI=FALSE,type="dop")

lcsdiffdistrprodCI

#The residualized change score model requires user inputs for the following:

#m2 ~ X*m1 value where X is the regression slope estimate of M2 on M1.

#y2 ~ X*y1 value where X is the regression slope estimate of Y2 on Y1.

res_syntax <-

'

#Defining change in M as a function of M1 and M2

deltam =~ 1*m2

deltam ~~ deltam

deltam ~ intm*1

m2 ~ 1.0561*m1

m2 ~~ 0*m1

m2 ~~ 0*m2

m2 ~ 0*1

m1 ~ 1

#Defining the change in Y as a function of Y1 and Y2

deltay =~ 1*y2

deltay ~~ deltay

deltay ~ inty*1

y2 ~ 1.0680*y1

y2 ~~ 0*y1

y2 ~~ 0*y2

y2 ~ 0*1

y1 ~ 1

#Estimating the Pretest correlation between M1 and Y1 and Variance of X

m1 ~~ y1

# Estimated covariance between M1 and X and Y1 and X because these covariances may not be equal to zero especially if X is not a randomized experiment

# without these the model has 2 degrees of freedom (covariances are only constrained to zero) but ANCOVA model should start out as saturated and have 0 degrees of freedom

m1 ~~ x # these covariances may not be equal to zero especially if X is not a randomized experiment

x ~~ y1 # these covariances may not be equal to zero especially if X is not a randomized experiment

#Regression of change in M on X and pretest measures

deltam ~ am2x*x

#Regression of change in Y on X, change in M, and pretest measures

deltay ~ x + by2m2*deltam

#Estimate of mediated effect

med := am2x*by2m2

'

res <- lavaan::sem(model = res_syntax, data = mydata, fixed.x = FALSE)

summary(res, standardized=FALSE, fit.measures=TRUE)

lcsares<-res@ParTable[["est"]][20]

lcsbres<-res@ParTable[["est"]][22]

lcsseares<-res@ParTable[["se"]][20]

lcssebres<-res@ParTable[["se"]][22]

#Using the saved regression coefficients and standard errors;

lcsresdistrprodCI<-medci(lcsares,lcsbres,lcsseares,lcssebres,rho=0,alpha=0.05,plot=FALSE,plotCI=FALSE,type="dop")

lcsresdistrprodCI

cross_syntax <-

'

#Defining change in M as a function of M1 and M2

deltam =~ 1*m2

deltam ~~ deltam

deltam ~ 1

m2 ~ 0*m1

m2 ~~ 0*m1

m2 ~~ 0*m2

m2 ~ 0*1

m1 ~ 1

#Defining the change in Y as a function of Y1 and Y2

deltay =~ 1*y2

deltay ~~ deltay

deltay ~ 1

y2 ~ 0*y1

y2 ~~ 0*y1

y2 ~~ 0*y2

y2 ~ 0*1

y1 ~ 1

#Estimating the Pretest correlation between M1 and Y1 and Variance of X

m1 ~~ y1

# Estimated covariance between M1 and X and Y1 and X because these covariances may not be equal to zero especially if X is not a randomized experiment

# without these the model has 2 degrees of freedom (covariances are only constrained to zero) but ANCOVA model should start out as saturated and have 0 degrees of freedom

m1 ~~ x # these covariances may not be equal to zero especially if X is not a randomized experiment

x ~~ y1 # these covariances may not be equal to zero especially if X is not a randomized experiment

#Regression of change in M on X and pretest measures

deltam ~ am2x*x

#Regression of change in Y on X, change in M, and pretest measures

deltay ~ x + by2m2*deltam

#Estimate of mediated effect

med := am2x*by2m2

'

cross <- lavaan::sem(model = cross_syntax, data = mydata, fixed.x = FALSE)

summary(cross, standardized=FALSE, fit.measures=TRUE)

lcsacross<-cross@ParTable[["est"]][20]

lcsbcross<-cross@ParTable[["est"]][22]

lcsseacross<-cross@ParTable[["se"]][20]

lcssebcross<-cross@ParTable[["se"]][22]

#Using the saved regression coefficients and standard errors;

lcscrossdistrprodCI<-medci(lcsacross,lcsbcross,lcsseacross,lcssebcross,rho=0,alpha=0.05,plot=FALSE,plotCI=FALSE,type="dop")

lcscrossdistrprodCI

**References**

Akaike, H. (1973). Information theory and an extension of the maximum likelihood principle. In B. N. Petrov & F. Csaki (Eds.), *Second International Symposium on Information Theory* (pp. 267–281). Budapest: Akademiai Kiado.

Bentler, P. M. (1995). EQS structural equations program manual. Encino, CA: Multivariate Software.

Hu, L. T., & Bentler, P. M. (1999). Cutoff criteria for fit indexes in covariance structure analysis: Conventional criteria versus new alternatives. *Structural equation modeling: a multidisciplinary journal*, *6*(1), 1-55.

Raftery, A. (1995). Bayesian model selection in social re- search. *Sociological Methodology, 25*, 111–196.

West, S. G., Taylor, A. B., & Wu, W. (2012). Model fit and model selection in structural equation modeling . In R. H. Hoyle (Ed.), *Handbook of structural equation modeling* (pp. 209-231). New York, NY: The Guilford Press.
